# Biodiversity footprints of 151 popular dishes from around the world

**DOI:** 10.1371/journal.pone.0296492

**Published:** 2024-02-21

**Authors:** Elissa M. Y. Cheng, Carina M. L. Cheng, Jacqueline Choo, Yanyun Yan, Luis Roman Carrasco

**Affiliations:** 1 Department of Biological Sciences, National University of Singapore, Singapore, Singapore; 2 Department of Statistics and Data Science, National University of Singapore, Singapore, Singapore; University of British Columbia, CANADA

## Abstract

Habitat loss for food production is a key threat to global biodiversity. Despite the importance of dietary choices on our capacity to mitigate the on-going biodiversity crisis, unlike with specific ingredients or products, consumers have limited information on the biodiversity implications of choosing to eat a certain popular dish. Here we estimated the biodiversity footprints of 151 popular local dishes from around the world when globally and locally produced and after calorical content standardization. We find that specific ingredients (beef, legumes, rice) encroaching on biodiversity hotspots with already very high agricultural pressure (e.g. India) lead to high biodiversity footprint in the dishes. Examples of high-biodiversity-footprint popular dishes were beef dishes such as fraldinha (beef cut dish) originating from Brazil and legume dishes such as chana masala (chickpea curry) from India. Regardless of assuming locally or globally produced, feedlot or pasture livestock production, vegan and vegetarian dishes presented lower biodiversity footprints than dishes containing meat. Our results demonstrate the feasibility of analysing biodiversity footprint at the dish level across multiple countries, making sustainable eating decisions more accessible to consumers.

## Introduction

The world’s biodiversity is undergoing a sixth mass extinction event with the average vertebrate extinction rate 100 times higher than the background rate [1]. This unprecedented rate of species extinctions is primarily driven by habitat loss resulting from expanding agriculture [2]. With the rising human population and economic growth expected to increase the demand for food up to 70% by 2050, the problem will be further exacerbated [3].

Transitioning towards sustainable diets—diets that reconcile environmental protection with food security and a healthy lifestyle for present and future generations—has been identified as a critical condition to conserve the world’s extant biodiversity [4]. With the food consumption of an average household contributing 20–30% of their final environmental impacts (including ozone depletion, terrestrial acidification, freshwater eutrophication, abiotic resource depletion and ecotoxicity) [5], sustainable food systems are essential to ensure the economic, social-cultural, and environmental welfare for the future [4].

One critical step to promote sustainable consumption is traceability as it improves the transparency of environmental actions [6]. Traceability helps to tackle the opacity of agri-food commodities’ supply chains due to international trade [7], where impacts on biodiversity such as the loss of habitat associated with consumption of a product expands well beyond the place of consumption [8]. Allowing environmentally conscious consumers to understand the impacts of their food purchasing and consumption decisions on the environment could reduce their environmental footprint by focusing on more sustainable products. For instance, sustainability information increased the purchasing intention rate by 1.15 points for users looking directly for sustainable products but had no impacts on buyers not looking specifically for sustainable products [9].

Currently, however, consumers’ decisions are mostly driven by price, taste and health, with sustainability considerations still requiring transparent and regulated labels [10]. Although the proportion of consumers that actively buys green-labelled products is relatively small (17% in Europe with a typical profile of young educated women for instance for fish), there is a large proportion of latent consumers that are willing to potentially pay more for environmentally friendly products (75% in Europe) which could become actual purchasers if price, freshness and origin, align [11].

Despite the large role of food consumption on biodiversity declines [12, 13], there are major gaps in biodiversity impacts accounting and reporting for businesses [14] with only 10 out of the 465 environmental certificates compiled by Ecolabel indexes specifically mentioning biodiversity [15]. This reflects the low availability of biodiversity footprint information as a criterion for consumers to choose products as compared to other criteria such as carbon footprint which is much more prevalent in labelling [16]. A lack of information on environmental issues and knowledge deters environmentally conscious consumers from purchasing green products [17], resulting in a slow transition towards sustainable diets.

The assessment of biodiversity impacts for specific ingredients and diets has progressed recently. For instance, with the biodiversity damage potential method which consists in comparing the species richness in a land use allocated to food production with a natural land use in a given biome. The proportion of species richness reduction attributed to land conversion from natural habitat to agriculture is then multiplied by the area of land converted to obtain impact scores [18]. Using this method, the types of foods involved in a specific diet can then be linked to the tabulated species richness impacts [19]. Research has also identified cropland footprints of dietary choices in e.g. Australia and Sweden using biodiversity impact factors and species-area relationships [20] at the ecoregion level [21, 22]. Species-area relationships present however limitations as they cannot capture the effect of the fragmentation of the landscape on extinction probability [23, 24]. A rarely explored alternative for biodiversity footprint analysis, yet a backbone for the International Union for Conservation of Nature (IUCN) Red List analysis of extinction threat, is to ascertain the area of the range of individual species that has been lost [25]. The development of recent datasets that identify the encroachment of specific crops on the ranges of birds, mammals and amphibians [26] opens an opportunity for alternative approaches for biodiversity footprint estimation.

Even though the previous identification of the biodiversity footprint in ingredients and diets can be very helpful for consumers, consumption decisions are not only done at the ingredient level, but at the product, or even, at dish level. A mismatch between the levels of aggregation of information and the consumers’ purchasing decision needs can pose a barrier to the adoption of sustainable consumption. Recent analyses of 57,000 products assessing greenhouse gas emissions and land use impacts, among others, helps bridge the gap between primary ingredients and composite products that are actively purchased (e.g. the environmental impacts of pesto are calculated from its ingredients such as olive oil, basil, pine nuts, facilitating the purchasing decision of consumers that buy pesto directly) [27].

Following the analogy with composite products, a fundamental higher level of aggregation for consumption decisions are dishes. Individuals decide what to consume at a restaurant or cook at home at the dish level, rather that at the primary ingredients level, following cultural and social factors, e.g. an Indian household may consider chicken chaat (dish made of chicken, vegetables and spices) versus chana masala (chickpea curry), or, in Brazil a common choice could be fraldinha (beef cut popular for BBQ, “churrasco”) versus arroz carreteiro (rice with grilled meat dish). Our knowledge of the biodiversity footprint at the dish level across multiple countries is limited, even though it could be very useful to simplify the choices of environmentally conscious consumers.

In the context of low biodiversity footprint information at the dish level across multiple countries, here we aim to calculate the biodiversity footprints of 151 popular dishes from around the world as an illustration. We do this assuming both locally and globally produced scenarios and under assumptions of feedlot-grown and pasture-grown livestock. We use recent biodiversity footprint databases of crops that are based on the concept of species ranges encroached by specific crops [26].

## Methods

### Overview

We considered popular dishes from countries that were in the top 25 in terms of gross domestic product in 2019. Dishes were selected from Cable News Network and when a list of dishes was not available for a given country, Taste Atlas was used instead [28, 29]. A total of 151 popular dishes were selected. The dishes were grouped into three categories: vegan, vegetarian, and dishes containing meat. Vegan dishes do not contain any animal-derived products, vegetarian dishes do not contain meat but include animal products such as milk and egg. Dishes containing meat include dishes with meat, poultry or fish.

The biodiversity footprint was calculated using three biodiversity indicators, namely species richness, threatened species richness, and range rarity affected by converting natural habitat to cropland or pastureland. Additionally, we considered four scenarios, feedlot-grown and locally produced, feedlot-grown and globally produced, pasture-grown and locally produced, and pasture-grown and globally produced. In the globally produced scenarios, biodiversity footprint was calculated based on the global distribution of species and crops, while the locally produced scenarios were calculated at the country level.

The biodiversity footprint of each of the ingredients composing the popular dishes after a calorical contribution normalization was assessed. This was done using biodiversity indexes [29] which analysed the agricultural footprint of individual crops and pasture on three taxa (mammals, birds, and amphibians). For livestock ingredients, they were translated to the crop and pasture inputs necessary to produce the livestock ingredient before using the biodiversity indexes. All data analysis and figures were produced in R version 4.1.1 [30].

### Biodiversity footprint of ingredients

The biodiversity footprint per unit of local crop yield (biodiversity footprint Mg^-1^) was calculated using the abovementioned biodiversity indicators [26]. The biodiversity footprint related to a crop or pasture area was the number of species, number of threatened species, or range rarity affected by converting natural habitat to cropland or pastureland. Biodiversity footprint per: (i) unit of local crop yield; and (ii) across agricultural areas datasets were used to calculate the biodiversity footprint of each dish ingredient. Dataset (i) was used for calculating the biodiversity footprint associated with a crop while dataset (ii) was used to calculate the biodiversity footprint related to pasture. Agricultural crops distributions were based on 175 *ca*. year 2000’s global crop-specific maps [31].

The distribution of biodiversity footprint values for a given crop or pasture and country across the raster cells of the country where the crop or pasture was distributed was presented through percentiles. We used the 50^th^ percentile data for each ingredient. In addition, outer fences were calculated by multiplying the interquartile range by three to identify extreme outliers in the datasets. The three crops identified as outliers were excluded and replaced with other crops within the fences when applicable (S1 Table).

To determine the biodiversity footprint of each dish, the biodiversity footprints of its ingredients were summed up. To calculate the biodiversity footprint of each crop ingredient in turn, we used:

$${(Biodiversity\;footprint\;of\;a\;crop\;ingredient=(W}_{c}∙F_{c}),$$ (1)

where *W*_*c*_ and *F*_*c*_ represent the fresh weight of a given crop *c* ingredient (in Mg) in the recipe of a dish and the biodiversity footprint of *c* (in number of species Mg^-1^, number of threatened species Mg^-1^ or range rarity Mg^-1^), respectively. Ingredients were matched to the closest corresponding crops or pasture in the biodiversity footprint dataset (S2 Table).

In the case of processed ingredients, the biodiversity footprint of the raw ingredients (calculated using Eq (1)) was multiplied by a corresponding conversion factor (S3 Table) to obtain the biodiversity footprint before this was used as an ingredient in the calculation of the dish’s biodiversity footprint. The conversion factor was the fraction of ingredient which can be extracted from its raw product.

### Calorical standardization

To ensure that the biodiversity footprint calculations of all dishes were comparable, calories were kept at a constant level for standardization. For all dishes, calories were standardised to be 825 kcal per dish, assuming daily calorical needs of 2000 kcal and a breakfast of 350 calories with the remaining two meals at 825 kcal each. This was done through proportionally changing the amount of the ingredients based on the recipe while keeping their relative proportions constant. The calorical information for each ingredient was taken from the US Department of Agriculture [32]. For recipes where quantity instead of the ingredient’s weight was stated, the weight was assumed as that included in the dataset [32]. In addition, when measurements of the ingredients were not readily convertible to the weights presented by US Department of Agriculture, conversion of the ingredients’ weights were done manually following guidelines, e.g. when fruits, eggs or bread slices were specified in units, they were converted to weight using the tables in the guidelines [33].

### Dishes selection and recipes

In the selection of dishes from Cable News Network or Taste Atlas (S4 Table contains the links to the dishes for each country and their sources), dishes were excluded if not all of their ingredients were represented in the biodiversity footprint dataset with the exception of salt that was not assessed for land use impacts. Countries for which Taste Atlas was the source of dishes were searched using the “dishes” tab followed by the “Selection” tab. The first 120 dishes were considered for countries with more than 100 entries. 151 dishes were eventually selected. The ingredients for each dish were estimated based on recipes in RecipeDB, a structured recipe database for global dishes, when available [34] or using alternative sources (S5 Table contains the recipes of each dish and its source). Processed ingredients such as tomato sauce used in the recipes were broken down into their raw ingredients sourcing additional recipes (S6 Table). One-way ANOVA was used to compare the biodiversity footprint between different categories of dishes (vegan, vegetarian and dishes containing meat). Tukey tests were used to correct for multiple comparisons.

### Scenarios

The biodiversity footprint of all dishes was assessed in both locally and globally produced scenarios. In the locally produced scenario, biodiversity footprint data from the selected country the dish was typical of was used. For the globally produced scenario, the global distribution of the crop was used to obtain a global biodiversity footprint without considering countries’ borders.

Dishes containing meat from livestock could have been produced using extensive methods based on pasture feeding or using intensive feedlot methods. We thus considered these methods as separate scenarios (pasture-grown and feedlot-grown scenarios) using the dataset that considers biodiversity footprint per unit of area of pasture and biodiversity footprint per ton of crop produced respectively [26]. The ingredients’ biodiversity footprint per kcal per gram under all three biodiversity indicators were also calculated.

For the feedlot-grown livestock, the biodiversity footprint of the livestock was calculated through the corresponding weights of crops constituting the livestock’s feed (S7 Table). The crop weights were obtained after multiplying the conversion ratio of the respective feeds with the weight of the livestock ingredient. The conversion ratio is the ratio of feed weight consumed to weight of livestock ingredient produced. After substitution of the livestock ingredient with its respective feeds, the biodiversity footprint of the livestock ingredients was calculated as the sum of the biodiversity footprints of the crops. Under the pasture-grown scenario, the biodiversity footprint of a livestock ingredient was calculated as:

$$Biodiversity\;footprint\;of\;a\;livestock\;ingredient=\frac{L_{m}∙F_{m\;}∙W_{m}}{C_{m}},$$ (2)

where *L*_*m*_ is the biodiversity footprint of the pasture to feed livestock *m* (in number of species ha^-1^, number of threatened species ha^-1^ or range rarity ha^-1^ respectively for each metric considered); *F*_*m*_ is the pasture area (in ha) required to produce one head of *m* (S8 Table); *W*_*m*_ is the weight of the livestock ingredient required in a given dish; and *C*_*m*_ is the carcass weight of one head of the livestock respectively (S9 Table). Pasture areas required to produce a head of livestock were based on countries or regions where the respective livestock production was the highest (S8 Table). The rationale for this assumption is that countries with large production pasture-fed livestock will present professional production systems in which the utilized area would be optimized, being a conservative estimate.

### Ambiguous ingredient types and measurements

When an ingredient was specified in a generic manner, the top ingredient of that type in terms of global production was assumed. For instance, soybean oil was assumed when only “oil” was mentioned in the recipe. White sugar was chosen for unspecified sugar type. To produce sugar, sugar beet was assumed as the sugar crop for all countries except tropical countries where sugar cane was assumed. Black pepper was selected as the pepper type and milk was assumed to be cow’s milk.

Standardisations of the recipes’ measurements were also made when weights were not specified. In recipes which stated “a pinch” as a form of measurement, they were standardised to be 0.5 g. Similarly, the amount of oil used for stir-frying was taken to be 1 tablespoon ~14 g per serving of food. Ingredients to be listed as added to taste were omitted. For deep-fried food, only the nutritional data of oil absorbed during the frying process was analysed. The amount of oil absorbed of the deep-fried food (S10 Table) was estimated as a proportion of the total weight of the food. In recipes where yeast was used, it was substituted with sourdough starter which was converted to flour and water [35].

## Results

### Biodiversity footprint of dishes

The biodiversity footprints of dishes containing meat were significantly higher than those of vegetarian and vegan dishes under all the locally and globally produced scenarios and all biodiversity footprint metrics compared except range rarity at the globally produced scenario (Table 1 shows the differences and S11 and S12 Tables the biodiversity footprint scores for each dish under the pasture-grown and feedlot-grown scenarios). Conversely, we failed to find a significant difference between vegan and vegetarian dishes (Table 1).

**Table 1:** Comparison of mean effects of dish type on different metrics of biodiversity footprints under the local vs. global production scenarios. The difference of the means corresponds to a one-way ANOVA and the p-values have been adjusted using Tukey (p-adj). Bold font denotes significant results.

	difference	lower	upper	p-adj
*Effect of dish type on local species richness*				
**Contains meat-Vegetarian**	**0.028**	**0.009**	**0.046**	**0.001**
Vegan-Vegetarian	-0.001	-0.018	0.015	0.979
**Vegan-Contains meat**	**-0.029**	**-0.043**	**-0.015**	**<0.001**
*Effect of dish type on local threatened species richness*				
**Contains meat-Vegetarian**	**0.001**	**0.000205**	**2.01·10** ^**−03**^	**0.012**
Vegan-Vegetarian	0.000	-0.00039	1.21·10^−03^	0.448
**Vegan-Contains meat**	**-0.001**	**-0.00137**	**-1.88·10** ^**−05**^	**0.042**
*Effect of dish type on local range rarity species richness*				
**Contains meat-Vegetarian**	**1.93·10** ^**−10**^	**6.89·10** ^**−11**^	**3.18·10** ^**−10**^	**0.001**
Vegan-Vegetarian	2.57·10^−12^	-1.08·10^−10^	1.14·10^−10^	0.998
**Vegan-Contains meat**	**-1.91·10** ^**−10**^	**-2.84E-** ^**10**^	**-9.74·10** ^**−11**^	**<0.001**
*Effect of dish type on global species richness*				
**Contains meat-Vegetarian**	**0.014**	**0.002**	**0.025**	**0.013**
Vegan-Vegetarian	-0.004	-0.014	0.007	0.687
**Vegan-Contains meat**	**-0.017**	**-0.026**	**-0.009**	**<0.001**
*Effect of dish type on global threatened species richness*				
Contains meat-Vegetarian	0.001	-9.46·10^−05^	0.001	0.111
Vegan-Vegetarian	0.000	-4.16·10^−04^	0.001	0.788
Vegan-Contains meat	0.000	-8.76·10^−04^	9.34·10^−05^	0.139
*Effect of dish type on global range rarity species richness*				
**Contains meat-Vegetarian**	**4.69·10** ^**−11**^	**1.24·10** ^**−12**^	**9.26·10** ^**−11**^	**0.043**
Vegan-Vegetarian	1.76·10^−12^	-3.90·10^−11^	4.25·10^−11^	0.994
**Vegan-Contains meat**	**-4.52·10** ^**−11**^	**-7.95·10** ^**−11**^	**-1.09·10** ^**−11**^	**0.006**

### Ingredients in high and low biodiversity footprint dishes

**Fig 1:**
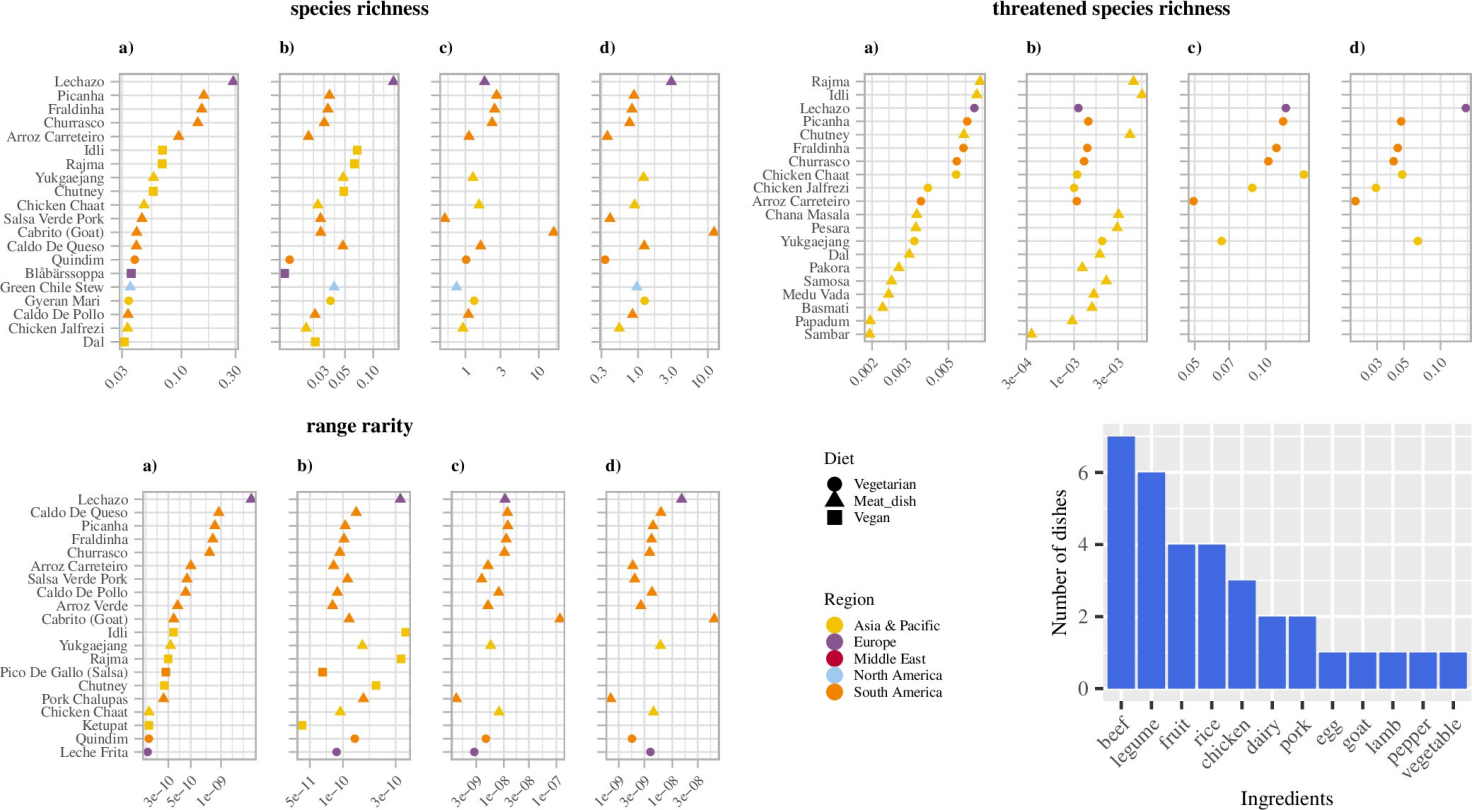
Top 20 dishes with the highest biodiversity footprint (per Mg) for all three biodiversity indicators. Scenario a) feedlot-grown locally produced, b) feedlot-grown globally produced, c) pasture-grown locally produced, and d) pasture-grown globally produced. Plot symbols and colours represent diet and dishes’ region of origin, respectively. Ingredients in the bar chart correspond to the main ingredient in terms of weight in a dish in the top 20 dishes with highest biodiversity footprints.

**Fig 2:**
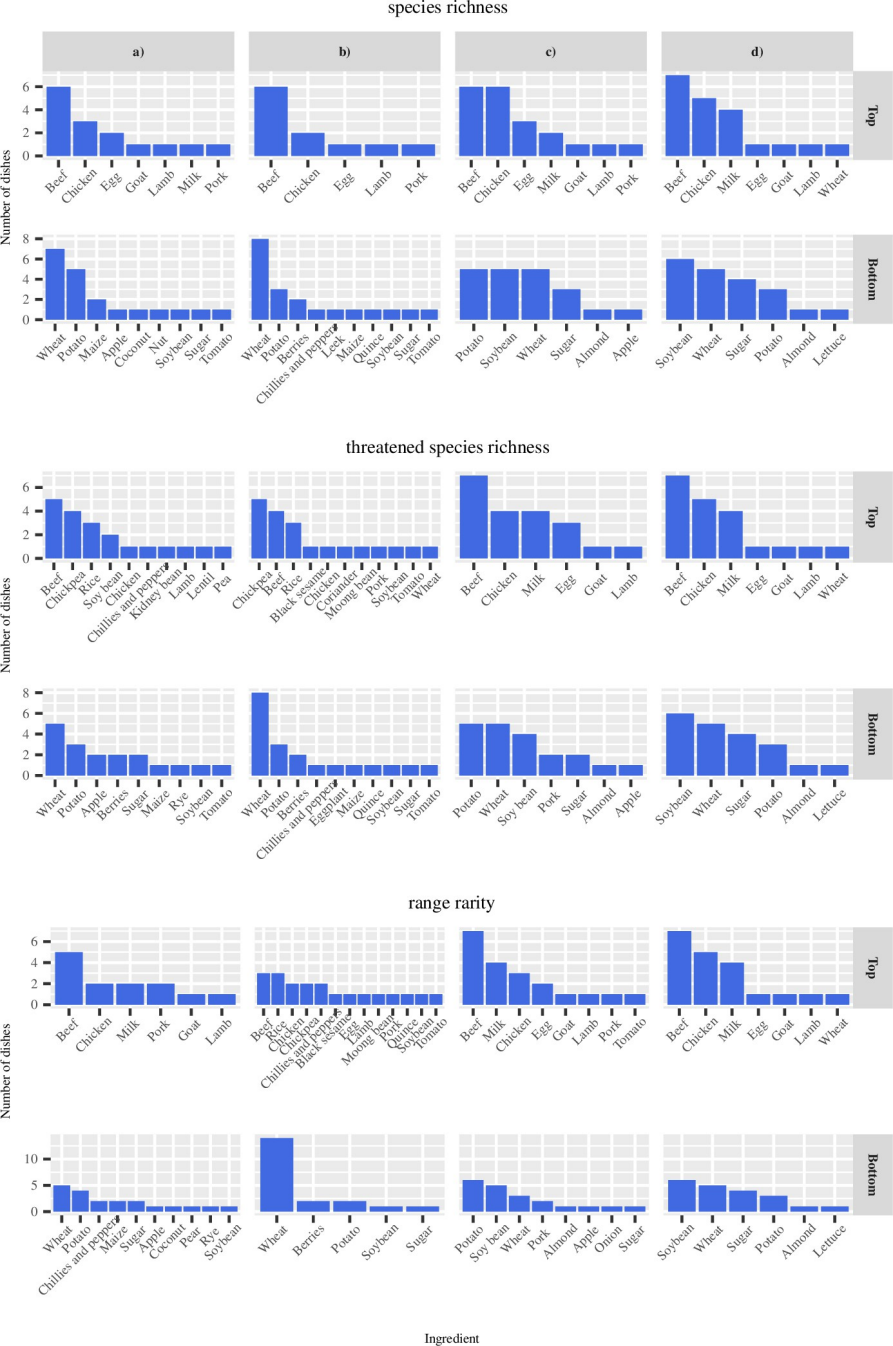
Main ingredients in terms of weight of the top and bottom 20 dishes. Scenario a) feedlot-grown locally produced, b) feedlot-grown globally produced, c) pasture-grown locally produced, and d) pasture-grown globally produced.

For all three biodiversity indicators, dishes with beef as the main ingredient such as fraldinha (traditional meat cut from Brazil) took up a large proportion of the top 20 dishes in terms of biodiversity footprint in all four scenarios (Figs 1 and 2). This result was explained by beef having a high biodiversity footprint per kcal per gram (S13 Table) which makes the mean of the biodiversity footprint of the dishes containing them the highest.

Besides beef, dishes with chicken, rice, or legumes as the main ingredient also took up a large proportion in the top 20 dishes in terms of biodiversity footprint (Fig 1). Rice and legumes were ranked higher under feedlot-grown scenarios for threatened species and range rarity biodiversity indicators (Fig 2). This was explained by the above-average biodiversity footprint per kcal per gram of legumes under threatened species richness in both locally and globally produced scenarios (S13 Table). High-biodiversity-footprint chicken, rice, and legume dishes tended to be from India and included chicken jalfrezi (type of tomato-based chicken curry), chicken chaat, chana masala, idli (savoury rice cake), and rajma (red kidney beans curry) (Fig 2).

**Fig 3:**
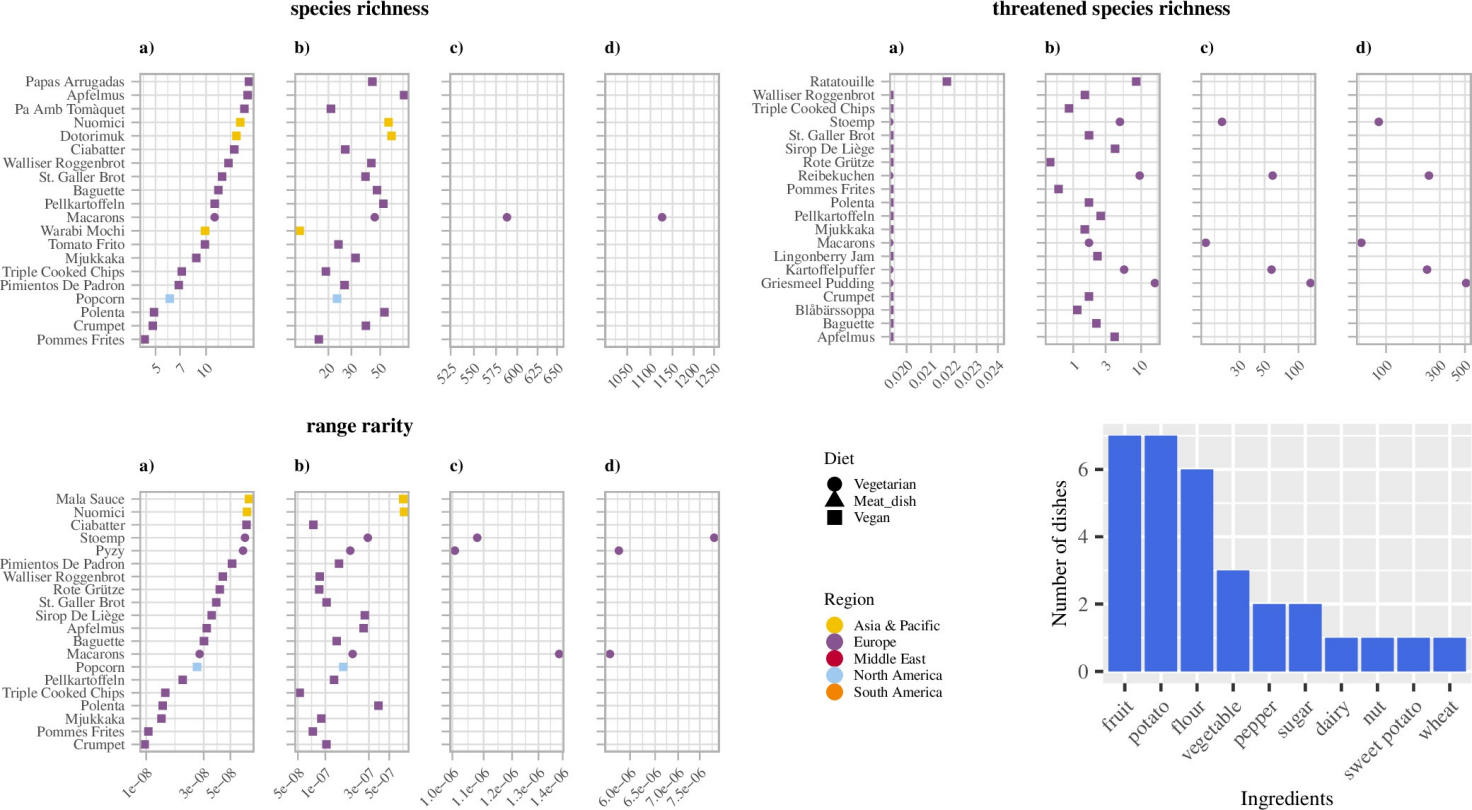
Bottom 20 dishes with the lowest biodiversity footprint (per 10 Gg) for all three biodiversity indicators. Scenario a) feedlot-grown locally produced, b) feedlot-grown globally produced, c) pasture-grown locally produced, and d) pasture-grown globally produced. Plot symbols and colours represent diet and dishes’ region of origin, respectively. Missing values in panels c) and d) occur for vegan dishes for which the pasture-grown scenarios are not relevant.

Among dishes with the lowest biodiversity footprint, starchy foods comprising potato and wheat (e.g. mantou, Chinese steamed bun) were represented in all four scenarios for all three biodiversity indicators (Figs 2 and 3). These results are partly explained by the lower weights of these dishes’ with below-average biodiversity footprint per kcal per gram under all indicators in both the locally and globally produced scenarios (S13 Table).

### Locally produced versus globally produced scenarios

Comparing the globally with the locally produced scenarios, the ranking in biodiversity footprint of beef dishes dropped under species richness and threatened species richness indicators while the rankings of egg and chicken dishes increased under all three biodiversity indicators. Similarly, under the species richness indicator for the pasture-grown scenario, dishes with chicken as the main ingredient took up a large proportion of the top dishes in the locally produced scenario, unlike the globally produced scenario (Fig 2). Under the feedlot-grown scenario for range rarity, dishes with legumes and rice took up a larger proportion of the top dishes in the globally produced scenario compared with the locally produced scenario (Fig 2).

## Discussion

Dishes with beef as the main ingredient (e.g. picanha, fraldinha (both beef cuts), chili con carne (spicy stew with chili peppers, beef and beans), and beef tartare (raw ground beef dish) were frequently top dishes in terms of biodiversity footprint under all three biodiversity indicators in all scenarios. In addition, we found a consistent significantly lower biodiversity footprint for vegan and vegetarian dishes compared to dishes containing meat. These results agree with previous studies finding a lower environmental impact associated with meatless diets [36]. The high biodiversity footprint associated with feedlot-grown beef is explained because cows have a less efficient and below-average feed conversion ratio as compared to the other animals [37]. For pasture-grown beef, it is also a result of cows requiring a large grazing area per unit of meat produced.

Under the locally produced scenario, the fact that the dishes originated from Brazil, further explained the dishes’ high biodiversity footprint. This result is explained by Brazil playing a major role in global markets for animal feeds such as soy [38]. Furthermore, following the rise in demand for animal feed in the late 1990s, the production of such feeds has since expanded into biodiversity hotspots such as the Cerrado which is not part of the Soy Moratorium and remains unprotected from land use change through soy agriculture [39]. This is in addition to the Amazon Forest which is greatly affected by land-use change from animal feed agriculture [40, 41]. For pasture-grown beef dishes, the high biodiversity footprint is likely due to Brazil’s land conversion within its biodiversity hotspots such as Cerrado and the Amazon Forest into pasturelands mainly to produce beef [42, 43]. This situation is further exacerbated by Brazil being home to the world’s highest number of endemic species [44] which explains the high number of threatened species affected by dishes containing beef produced in Brazil.

Lechazo, lamb dish from Spain, was the dish with the highest biodiversity footprint. This result is due to the poor conversion efficiency of lamb combined with a relatively high area of pasture needed for its production [45]. The fact that the dish is from Spain is however due to not having lamb dishes from Brazil, Australia or Thailand. Lamb produced in these countries would achieve a much higher biodiversity footprint as it presents the highest biodiversity footprint per gram and calorie (S13 Table).

The bottom dishes in terms of biodiversity footprint included mainly starchy dishes such as baguette (French loaf of bread), warabi mochi (dessert made of starch from the bracken root), and pyzy (potato dumpling) under all three biodiversity indicators and in all four scenarios. For the locally produced scenario, these starchy foods with low biodiversity footprints mainly originated from countries in temperate regions such as Poland and France. Temperate regions have lower species richness than tropical regions and shelter species with greater range sizes as greater temperature variability selects for species with wider ranges [46]. The greater range would also reduce species’ vulnerability to anthropogenic activities and decrease species’ susceptibility to extinction [47].

Surprisingly, besides beef, dishes with chicken, rice, or legumes as the main ingredient had also high biodiversity footprints. These results are explained by these dishes mainly originating from India and Mexico. Land conservation to agriculture has occurred in India at the expense of sup alpine forests, such as the Indian Himalaya Region [48], which is another global biodiversity hotspot. Similarly, Mexico presents pastureland expansion at the expense of its tropical forest biome ecoregions [49]. India is both a top producer of legumes and one of the mega-diverse countries of the world containing 7–8% of global discovered species [50]. In addition, chickpeas, the legume included in approximately 60% of the high biodiversity footprint legume dishes is cultivated in the southern parts of the Western Ghats [51], another biodiversity hotspot. For rice dishes (e.g. idli, ketupat (Javanese rice cake), and basmati (Indian rice dish)), the high global biodiversity footprints could be attributed to 60% of rice being produced in the tropics, such as in India, and Indonesia. The tropics select for species with smaller range sizes due to lower latitude and temperature seasonality [46], resulting in higher range rarity.

Our results highlight the urgency for policy formulation regarding agricultural practices in countries such as India and Brazil where high biodiversity footprint dishes originated from. Some policy formulations could include taxes on dishes with high biodiversity footprint or their related ingredients [19] such as beef while subsidizing other alternative sources of protein. With price being a concern towards green consumption especially during the initial stages of adoption, lower prices for dishes with a low biodiversity footprint could help to better ease the conversion to low biodiversity footprint dishes [52]. Our results could also encourage the development of restoration projects in areas where the highest biodiversity footprints occur, e.g. restoration projects to increase connectivity have been identified as a priority in the Atlantic Forest in Brazil [53]. Other suitable interventions could be land sparing via agricultural intensification, yet these implications need to be observed from the perspective of sustainable intensification to prevent further impacts on biodiversity [54].

Our analyses present several limitations. The biodiversity footprint data of pasture obtained from Beyer and Manica [26] were general and not specific to different livestock. This might lead to inaccuracies regarding the biodiversity footprints associated with the respective pasture-grown livestock ingredients. Although our approach opens an alternative way to estimate biodiversity footprint of food production based on IUCN Red List approaches for extinction risk estimation, it still has room to be further developed. For instance, no distinction was made between species that survive in agricultural land vs. species that have natural habitat requirements and only mammals, birds and amphibians were considered. Similarly, the ranges of species could be refined to consider only area of suitable habitat [25].

Our analyses were standardised based on calorie content but this does not necessarily reflect nutritional needs. This standardization contributes to the result by which low biodiversity footprint dishes identified are mostly carbohydrates with low nutritional content. Future analyses standardised on nutritional needs such as protein, essential lipids, minerals, and vitamins is needed to better reveal nutritious dishes with low biodiversity footprint for a step towards sustainable diets.

Our list of dishes is far from exhaustive and not likely to be representative of the dishes of the world. One factor is that we focused on dishes from countries that are top 25 in terms of GDP. This led to no dishes from Africa. In addition, due to constraints resulting in the complexity of intermediate products used in recipes, we did not exhaustively analyse all available dishes in a given country. Our dish sample needs thus to be regarded as an illustration of the potential of the methods to be applied to a variety of dishes. Our limited sample of dishes highlights the need to develop datasets of biodiversity footprint of main ingredients and products used in dishes worldwide.

While this study used the world biodiversity footprint in globally produced scenarios to consider the possibility of global trade, future research could consider using the biodiversity footprints of specific countries that the ingredients were sourced from considering actual trade links with their embedded biodiversity impacts [8]. Finally, dishes recipes can present high variability which could lead to different results.

## Conclusion

Our study attempts to provide a higher level of aggregation of units of consumption, i.e. dishes, with regards to their biodiversity footprint across multiple countries. Overall, our results point to the importance of certain ingredients and regions of production in the overall biodiversity footprint of a dish, namely dishes with high biodiversity footprints include beef dishes such as fraldinha (beef cut) originating from Brazil and legume dishes such as chana masala (chickpea curry) from India. On the other hand, vegan and vegetarian dishes tend to have low biodiversity footprints. Comparisons between locally and globally produced scenarios suggests the importance of dish’s country of origin to determine the source of production with a lower biodiversity footprint where India was observed to be involved with the production of mostly high biodiversity footprint dishes with biodiversity impacts driven by ingredients (e.g. rice, legumes, chicken) that are not commonly flagged as having a high environmental footprint. Moving towards the estimation of dishes’ biodiversity footprints across multiple countries can help to fill consumers’ knowledge gap in an accessible manner. A combination of consumer awareness to facilitate a transition towards sustainable diets is imperative to mitigate the large impacts of food production on biodiversity.

## Supporting information

S1 Table Outliers and their replacement crops datasets used in the calculation of biodiversity footprint of dishes. (DOCX)

S2 Table Correspondence between ingredients and crop and pasture categories in the biodiversity footprint dataset. (XLSX)

S3 Table Conversion factors of ingredients derived from their respective raw product. For brown sugar, it was calculated as the sum of proportion of molasses in brown sugar multiplied by conversion factor of molasses and the proportion of white sugar in brown sugar multiplied by the conversion factor of white sugar.
(DOCX)

S4 Table Links of articles used to select popular dishes from countries using Cable News Network. (DOCX)

S5 Table Recipes of all dishes considered per country, their weight in each recipe and calories. (XLSX)

S6 Table Recipes and nutrient data for processed ingredients found in main recipes. These ingredients were standardised to be 100 g or 1 serving size. The calories (kcal) of the corresponding ingredients were based on US Department of Agriculture.—Weight and nutritional profile of water and salt were omitted.
(DOCX)

S7 Table Feed composition and conversion ratio of the various livestock ingredients. Chicken and eggs were assumed to have the same feed. Forage was assumed to be wheat and alfalfa for dairy cattle and lamb respectively.
(DOCX)

S8 Table Grazing area of the livestock which the livestock ingredient was obtained from in the corresponding countries. (DOCX)

S9 Table Carcass weights (kg) of the respective livestock. (DOCX)

S10 Table Deep-fried food products and their respective oil absorbed during frying as a proportion of their weight. (DOCX)

S11 Table Biodiversity footprints for vegan, vegetarian and dishes containing meat in feedlot-grown scenario in both locally and globally produced scenario under species richness, threatened species richness and range rarity indictors. The dishes are ranked according to species richness indicator.
(DOCX)

S12 Table Biodiversity footprints for vegetarian and dishes containing meat in pasture-grown scenario in both locally and globally produced scenario under species richness, threatened species richness and range rarity indictors. The dishes are ranked according to species richness indicator.
(DOCX)

S13 Table Average biodiversity footprint per kcal per gram for each country–ingredient combinations considered under the species richness, threatened species richness and range rarity metrics. (XLSX)

S1 Text Supporting references. (DOCX)
