# Supplementary material for: Biodiversity footprints of 151 popular dishes from around the world
Source: PLoS One. 2024 Feb 21;19(2):e0296492. doi: 10.1371/journal.pone.0296492 (PMC10880993; doi:10.1371/journal.pone.0296492)
Supplement: S1 Table — (DOCX) [file pone.0296492.s001.docx]

| **Outliers** | **Replacement Crop** |  |
| --- | --- | --- |
| Popcorn | Maize, green | (Suleiman et al., 2015) |
| Cloves | Spices, nes | (Srivastava, 1993) |
| Nutmeg, mace and cardamoms | Spices, nes | (Rema and Krishnamoorthy, 2012) |
