# Supplementary material for: Biodiversity footprints of 151 popular dishes from around the world
Source: PLoS One. 2024 Feb 21;19(2):e0296492. doi: 10.1371/journal.pone.0296492 (PMC10880993; doi:10.1371/journal.pone.0296492)
Supplement: S3 Table — For brown sugar, it was calculated as the sum of proportion of molasses in brown sugar multiplied by conversion factor of molasses and the proportion of white sugar in brown sugar multiplied by the conversion factor of white sugar. (DOCX) [file pone.0296492.s003.docx]

| **Ingredient** | **Conversion Factor** |  |
| --- | --- | --- |
| **Flour** |  |  |
| All purpose | 0.800 | (LCAfood-conference, 2007a) |
| Almond | 1.00 | (Alderson, 2014) |
| Cake | 0.800 | (LCAfood-conference, 2007a) |
| Chickpea | 1.00 | (Alderson, 2014) |
| Glutinous | 1.00 | (Alderson, 2014) |
| Rice | 1.00 | (Alderson, 2014) |
| Rye | 0.700 | (LCAfood-conference, 2007a) |
| Wheat | 0.800 | (LCAfood-conference, 2007a) |
| Potato | 0.200 | (Walker, 1993) |
| Semolina | 1.000 | (Alderson, 2014) |
| Soybean | 0.880 | (Inglett, 2012) |
| **Meal** |  |  |
| Soybean | 0.822 | (Dalgaard et al., 2007; LCAfood-conference, 2007f) |
| Corn | 0.906 | (Samira, 2021) |
| **Starch** |  |  |
| Corn | 1.00 | (Alderson, 2014) |
| Potato | 0.950 | (Bergthaller et al., 1999) |
| Sweet potato | 0.250 | (Kang and Priyadarshan, 2008) |
| **Juice** |  |  |
| Lemon | 0.303 | (Moufida and Marzouk, 2003) |
| Lime | 0.416 | (Thirst Quenching Lime, 1983) |
| **Peel or Flake** |  |  |
| Lemon | 0.400 | (Ahmad et al., 2016) |
| Coconut | 0.160 | (Gaston O. Adoyo, 2021) |
| **Zest** |  |  |
| Lemon | 0.275 | (Velasco-Arango et al., 2020) |
| **Pulp** |  |  |
| Tamarind | 0.550 | (Kumar and Bhattacharya, 2008) |
| **Oil** |  |  |
| Rapeseed | 0.416 | (LCAfood-conference, 2007f) |
| Sesame | 0.500 | (Addison, 2021) |
| Olive | 0.400 | (Vossen, 2007) |
| Peanut | 0.348 | (Tsao et al., 2021) |
| Corn | 0.220 | (Moreau et al., 2005) |
| Soybean | 0.200 | (Özbek and Ergönül, 2020) |
| Sunflower | 0.376 | (Tsao *et al.*, 2021) |
| Coconut | 0.330 | (Mike Foale, 2011) |
| Grape seed | 0.200 | (Martin et al., 2020) |
| **Sugar** |  |  |
| Light brown | 0.144 | (LCAfood-conference, 2007e; S.) |
| White | 0.137 | (LCAfood-conference, 2007e) |
| Molasses | 0.240 | (LCAfood-conference, 2007e) |
| Cane | 0.103 | (Yuttitham et al., 2011) |
| **Powder** |  |  |
| Garlic | 0.388 | (Samira, 2020) |
| Turmeric | 0.085 | (Hirun et al., 2014) |
| Acorn | 0.418 | (Moon et al., 2013) |
| Asafoetida | 0.760 | (Pearson, 1910) |
| **Paste** |  |  |
| Tomato | 0.250 | (Scherer and Pfister, 2016) |
| **Milk (Cream)** |  | (Mike, 2011) |
| Coconut | 0.600 | (Chetachukwu et al., 2018) |
| **Seed** |  |  |
| Coriander | 0.768 | (Kumar et al., 2015; Shahwar et al., 2012) |
| **Dried spices** |  |  |
| Parsley | 0.186 | (Soysal, 2004) |
| Oregano | 0.087 | (Figiel et al., 2010) |
| Rosemary | 0.236 | (Bensebia and Allia, 2016) |
| Basil | 0.173 | (Özcan et al., 2005) |
| Curry leaf | 0.040 | (Sakhale et al., 2007) |
| **Pepper** |  |  |
| Black | 0.350 | (Aziz et al., 2019) |
| White | 0.265 | (Aziz *et al.*, 2019) |
| **Nuts** |  |  |
| Deshelled peanut | 0.725 | (Varma et al., 2020) |
| Blanched almond | 0.940 | (Arena et al., 2010) |
| Deshelled almond | 0.300 | (U.S. Department of Agriculture, 1992) |
| **Livestock Feed** |  |  |
| Hay | 0.295 | (Siles et al., 2015) |
