# Supplementary material for: Biodiversity footprints of 151 popular dishes from around the world
Source: PLoS One. 2024 Feb 21;19(2):e0296492. doi: 10.1371/journal.pone.0296492 (PMC10880993; doi:10.1371/journal.pone.0296492)
Supplement: S4 Table — (DOCX) [file pone.0296492.s004.docx]

| Country | Continent | Number of dishes selected | Vegan | Vegetarian | Contains meat | Dishes considered | Source |
| --- | --- | --- | --- | --- | --- | --- | --- |
| Australia | Oceania | 1 | 1 |  |  | 40 | <https://edition.cnn.com/travel/article/australian-food/index.html> |
| Belgium | Europe | 3 | 2 | 1 |  | 103 | https://www.tasteatlas.com/belgium |
| Brazil | Americas | 6 | 1 | 1 | 4 | 120 | https://www.tasteatlas.com/brazil |
| Canada | Americas | 1 |  |  | 1 | 71 | https://www.tasteatlas.com/canada |
| China | Asia | 15 | 5 | 1 | 9 | 32 | <https://edition.cnn.com/travel/article/best-chinese-food-dishes-to-try-cmd/index.html> |
| France | Europe | 4 | 3 | 1 |  | 120 | https://www.tasteatlas.com/france |
| Germany | Europe | 5 | 3 | 2 |  | 20 | <https://edition.cnn.com/travel/article/german-food/index.html> |
| India | Asia | 26 | 24 |  | 2 | 120 | https://www.tasteatlas.com/india |
| Indonesia | Asia | 1 | 1 |  |  | 40 | <https://edition.cnn.com/travel/article/40-indonesian-foods/index.html> |
| Italy | Europe | 6 | 6 |  |  | 120 | https://www.tasteatlas.com/italy |
| Japan | Asia | 6 | 5 |  | 1 | 25 | <https://edition.cnn.com/travel/article/japan-25-foods/index.html> |
| Mexico | Americas | 10 | 3 |  | 7 | 120 | https://www.tasteatlas.com/mexico |
| Netherlands | Europe | 1 |  | 1 |  | 117 | https://www.tasteatlas.com/netherlands |
| Poland | Europe | 8 |  | 5 | 3 | 15 | <https://edition.cnn.com/travel/article/best-polish-foods/index.html> |
| Russia | Europe | 4 | 1 | 1 | 2 | 64 | https://www.tasteatlas.com/russia |
| Saudi Arabia | Asia | 1 | 1 |  |  | 20 | <https://edition.cnn.com/travel/article/middle-east-food-dishes/index.html> |
| South Korea | Asia | 6 | 3 | 1 | 2 | 39 | <https://edition.cnn.com/travel/article/best-korean-dishes/index.html> |
| Spain | Europe | 22 | 11 | 8 | 3 | 120 | [https://edition.cnn.com/travel/article/essential-spanish-dishes/index.html; https://www.tasteatlas.com/spain](https://edition.cnn.com/travel/article/essential-spanish-dishes/index.html) |
| Sweden | Europe | 3 | 3 |  |  | 96 | https://www.tasteatlas.com/sweden |
| Switzerland | Europe | 2 | 2 |  |  | 66 | https://www.tasteatlas.com/switzerland |
| Turkey | Asia | 4 | 2 | 2 |  | 23 | <https://edition.cnn.com/travel/article/best-turkish-foods/index.html> |
| United Kingdom | Europe | 2 | 2 |  |  | 20 | <https://edition.cnn.com/travel/article/classic-british-food/index.html> |
| United States | Americas | 12 | 5 | 1 | 6 | 50 | <https://edition.cnn.com/travel/article/american-food-dishes/index.html> |
| Thailand | Asia | 1 | 1 |  |  | 120 | https://www.tasteatlas.com/thailand |
| Venezuela | Americas | 1 | 1 |  |  | 23 | https://www.tasteatlas.com/venezuela |
