# Supplementary material for: Biodiversity footprints of 151 popular dishes from around the world
Source: PLoS One. 2024 Feb 21;19(2):e0296492. doi: 10.1371/journal.pone.0296492 (PMC10880993; doi:10.1371/journal.pone.0296492)
Supplement: S6 Table — These ingredients were standardised to be 100 g or 1 serving size. The calories (kcal) of the corresponding ingredients were based on US Department of Agriculture.—Weight and nutritional profile of water and salt were omitted. (DOCX) [file pone.0296492.s006.docx]

| **Simply processed ingredient (100 g or 1 serving)** | **Ingredient** | **Weight per ingredient (g)** | **Calories per ingredient (kcal)** | **Total calories (kcal) per simply processed ingredient (100 g or 1 serving)** | **Recipe source** |
| --- | --- | --- | --- | --- | --- |
| **Beef Stock** | Pound Beef | 108 | 0.184 | 21.1 | (Ojakangas, 2020) |
|  | Onion | 4.70 | 1.69 |  |  |
|  | Carrot | 12.9 | 5.02 |  |  |
|  | Celery | 2.11 | 0.296 |  |  |
|  | Parsnip | 5.07 | 3.80 |  |  |
|  | Water | - | - |  |  |
|  | Parsley | 0.106 | 0.038 |  |  |
|  | Garlic | 0.158 | 8.94 |  |  |
|  | Dried Thyme | 0.300 | 0.303 |  |  |
|  | Bay Leaf | 0.0475 | 0.272 |  |  |
|  | Black Pepper | 0.211 | 0.530 |  |  |
|  |  |  |  |  |  |
| **Chicken Broth** | Chicken Bone | 95.8 | 215 | 239 | (Ojakangas, 2020) |
|  | Onion | 28.2 | 11.3 |  |  |
|  | Celery | 0.317 | 1.77 |  |  |
|  | Carrot | 25.7 | 10.5 |  |  |
|  |  |  |  |  |  |
| **Curry Powder** | Cumin | 40.0 | 150 | 256 | (Batra,2019bc) |
|  | Coriander | 24.0 | 5.51 |  |  |
|  | Turmeric | 14.7 | 57.1 |  |  |
|  | Red Pepper Flake | 2.80 | 9.53 |  |  |
|  | Mustard Seed | 3.33 | 5.08 |  |  |
|  | Ginger | 8.66 | 29.0 |  |  |
|  |  |  |  |  |  |
| **Dark Soy Sauce** | Soy Sauce | 19.0 | 2460 | 2470 | (Bill, 2019) |
|  | Molasses | 1.14 | 4.40 |  |  |
|  | White Sugar | 1.19 | 4.58 |  |  |
|  |  |  |  |  |  |
| **Pasta** | Egg | 78.7 | 112 | 540 | (Batra,2019at) |
|  | All Purpose Flour | 120 | 428 |  |  |
|  |  |  |  |  |  |
| **Peanut Butter** | Peanut | 100 | 567 | 592 | (Thomas, 2018) |
|  | Vegetable Oil | 2.80 | 24.8 |  |  |
|  |  |  |  |  |  |
| **Soy Sauce** | Soybean | 1812 | 8080 | 12940 | (Pinoy, 2021) |
|  | All Purpose Flour | 1360 | 4860 |  |  |
|  | Salt | - | - |  |  |
|  | Water | - | - |  |  |
|  |  |  |  |  |  |
| **Sourdough Starter** | Rye Flour | 33.3 | 133 | 445 | (Beranbaum, 2003) |
|  | Bread Flour | 100 | 312 |  |  |
|  | Water | - | - |  |  |
|  |  |  |  |  |  |
| **Garam Masala** | Black Pepper | 31.4 | 78.7 | 222 | (Rai, 2000) |
|  | Coriander Seed | 21.8 | 65.0 |  |  |
|  | Cardamom Pod | 12.1 | 37.7 |  |  |
|  | Cumin Seed | 7.95 | 29.8 |  |  |
|  | Cinnamon Stick | 5.30 | 11.2 |  |  |
|  |  |  |  |  |  |
| **Paneer** | Milk | 1790 | 1090 | 1100 | (Batra, 2019bg) |
|  | Lemon Juice | 44.6 | 9.82 |  |  |
|  |  |  |  |  |  |
| **Flour Tortilla** | All Purpose Flour | 37.5 | 137 | 172 | (Batra,2019as) |
|  | Salt | - | - |  |  |
|  | Lard | 3.69 | 30.4 |  |  |
|  | Water | - | - |  |  |
|  |  |  |  |  |  |
| **Corn Tortilla** | Water | - | - |  | (Batra,2019e) |
|  | Cornmeal | 9.81 | 36.3 | 168 |  |
|  | All Purpose Flour | 31.3 | 114 |  |  |
|  | Salt | - | - |  |  |
|  | Egg | 12.6 | 18.0 |  |  |
|  |  |  |  |  |  |
| **Lard** | Pork Fat | 442 | 823 | 823 | (Solomon and Martin, 2009) |
|  | Water | - | - |  |  |
|  |  |  |  |  |  |
| **Pepper Sauce** | Cayenne Pepper | 3.74 | 11.9 | 698 | (Batra, 2019be) |
|  | Cumin | 2.12 | 7.95 |  |  |
|  | Garlic | 2.12 | 3.16 |  |  |
|  | Salt | - | - |  |  |
|  | Olive Oil | 76.3 | 675 |  |  |
|  |  |  |  |  |  |
| **Raspberry Jam** | Raspberry | 37.9 | 19.7 | 258 | (Foody, 2022) |
|  | Lemon Juice | 1.174 | 0.259 |  |  |
|  | White Sugar | 61.6 | 238 |  |  |
|  |  |  |  |  |  |
| **Rice Noodle** | Rice Flour | 33.2 | 121.4 | 134 | (Judy, 2020) |
|  | Tapioca Starch | 2.64 | 9.90 |  |  |
|  | Salt | - | - |  |  |
|  | Water | - | - |  |  |
|  | Vegetable Oil | 0.765 | 2.82 |  |  |
|  |  |  |  |  |  |
| **Salsa** | Plum Tomato | 77.6 | 14.0 | 25.8 | (Batra, 2019bm) |
|  | Onion | 22.9 | 9.2 |  |  |
|  | Cilantro | 0.626 | 0.144 |  |  |
|  | Lime Juice | 6.31 | 1.58 |  |  |
|  | Garlic Clove | 0.626 | 0.932 |  |  |
|  | Salt | - | - |  |  |
|  |  |  |  |  |  |
| **Beef Broth** | Beef | 24.0 | 71.7 | 77.3 | (Batra,2019i) |
|  | Parsley | 0.159 | 0.0572 |  |  |
|  | Salt | - | - |  |  |
|  | Celery | 4.23 | 0.597 |  |  |
|  | Carrot | 6.45 | 2.64 |  |  |
|  | Onion | 5.81 | 2.33 |  |  |
|  |  |  |  |  |  |
| **Chicken Stock** | Chicken | 42.2 | 19.6 | 21.3 | (Batra,2019u) |
|  | Water | - | - |  |  |
|  | Ginger | 0.194 | 0.155 |  |  |
|  | Spring Onion | 3.30 | 1.06 |  |  |
|  | Garlic | 0.528 | 0.295 |  |  |
|  | Turmeric | 0.0484 | 0.176 |  |  |
|  | Salt | - | - |  |  |
|  |  |  |  |  |  |
| **Baguette** | All Purpose Flour | 7.09 | 25.8 | 36.1 | (Hadjiandreou, 2016) |
|  | Salt | - | - |  |  |
|  | Water | - | - |  |  |
|  | Sourdough Starter | 0.236 | 1.05 |  |  |
|  | Water | - | - |  |  |
|  | Bread Flour | 2.96 | 9.21 |  |  |
|  |  |  |  |  |  |
| **Whole Wheat Bread** | Whole Wheat Flour | 62.5 | 231 | 245 | (Hadjiandreou, 2016) |
|  | Salt | - | - |  |  |
|  | Sourdough Starter | 3.13 | 13.9 |  |  |
|  | Water | - | - |  |  |
|  |  |  |  |  |  |
| **White Bread** | All Purpose Flour | 126 | 460 | 672 | (Morton, 2019) |
|  | Sourdough Starter | 44.6 | 199 |  |  |
|  | Water | - | - |  |  |
|  | Salt | - | - |  |  |
|  | Semolina Flour | 3.48 | 12.5 |  |  |
|  |  |  |  |  |  |
| **Bread** | All Purpose Flour | 134 | 488 | 699.5 | (Morton, 2019) |
|  | Water | - | - |  |  |
|  | Sourdough Starter | 44.6 | 199 |  |  |
|  | Salt | - | - |  |  |
|  | Semolina Flour | 3.48 | 12.5 |  |  |
|  |  |  |  |  |  |
| **Hamburger Bun** | All Purpose Flour | 18.7 | 67.9 | 197 | (Dongre, 2020) |
|  | White Sugar | 3.15 | 12.2 |  |  |
|  | Sourdough Starter | 17.1 | 77.8 |  |  |
|  | Salt | - | - |  |  |
|  | Water | 22.1 | 0.0 |  |  |
|  | Egg | 6.29 | 8.99 |  |  |
|  | Olive Oil | 2.13 | 29.8 |  |  |
|  |  |  |  |  |  |
| **Wheat Noodle** | Salt | - | - |  | (Nyonya Cooking, 2021) |
|  | Vegetable Oil | 3.10 | 27.4 | 273 |  |
|  | Wheat Flour | 66.4 | 245 |  |  |
|  | Water | - | - |  |  |
|  |  |  |  |  |  |
| **Country Style Bread** | Sourdough Starter | 1.45 | 6.45 | 58.75 | (Amy, 2021) |
|  | Water | - | - |  |  |
|  | Wheat Flour | 0.723 | 32.1 |  |  |
|  | Bread Flour | 6.51 | 20.2 |  |  |
|  | Salt | - | - |  |  |
|  |  |  |  |  |  |
| **Bread Flour** | All Purpose Flour | 82.7 | 301 | 312 | (Marshall, 2021) |
|  | Vital Wheat Gluten | 8.55 | 31.5 |  |  |
|  |  |  |  |  |  |
| **Vital Wheat Gluten** | Wheat Flour | 118 | 435 | 435 | (Sayaslan, 2004) |
|  |  |  |  |  |  |
| **Bread Crumb** | All Purpose Flour | 253 | 921 | 1318 | (Morton, 2019; Stafford, 2020) |
|  | Sourdough Starter | 89.3 | 397 |  |  |
|  | Water | - | - |  |  |
|  | Salt | - | - |  |  |
|  |  |  |  |  |  |
| **Five Spice Powder** | Fennel Bulb | 41.4 | 143 | 563 | (Batra,2019x) |
|  | Black Pepper | 32.9 | 82.4 |  |  |
|  | Star Anise | 60.0 | 202 |  |  |
|  | Cinnamon | 46.4 | 115 |  |  |
|  | Clove | 7.50 | 20.5 |  |  |
|  |  |  |  |  |  |
| **Mixed Spice** | Nutmeg | 33.2 | 175 | 384 | (Batra,2019bd) |
|  | Cinnamon | 12.3 | 30.4 |  |  |
|  | Allspice | 28.4 | 74.9 |  |  |
|  | Mace | 16.1 | 76.6 |  |  |
|  | Clove | 9.95 | 27.2 |  |  |
|  |  |  |  |  |  |
| **Salt Pork** | Salt | - | - | 510 | (Cook.me Inc, 2019) |
|  | White Sugar | 5.6 | 21 |  |  |
|  | Pork Belly | 95 | 489 |  |  |
|  |  |  |  |  |  |
| **Tomato Onion Salsa** | Tomato | 18.5 | 2.97 | 174 | (Raichlen, 2010) |
|  | Onion | 9.17 | 3.67 |  |  |
|  | Yellow Bell Pepper | 15.5 | 4.18 |  |  |
|  | Parsley | 1.90 | 0.685 |  |  |
|  | Vegetable Oil | 18.2 | 161 |  |  |
|  | Salt | - | - |  |  |
|  | Lime Juice | 7.56 | 1.89 |  |  |
|  |  |  |  |  |  |
| **Green Pepper Sauce** | Canola Oil | 1.47 | 13.0 | 97.2 | (Joachim and Hoffman, 2000) |
|  | Onion | 8.98 | 3.59 |  |  |
|  | Garlic | 0.490 | 0.730 |  |  |
|  | All Purpose Flour | 2.55 | 9.29 |  |  |
|  | Cumin | 0.086 | 0.322 |  |  |
|  | Black Pepper | 27.0 | 67.8 |  |  |
|  | Vegetable Broth | 37.0 | 5.99 |  |  |
|  | Green Chili Pepper | 0.128 | 0.0510 |  |  |
|  | Dried Oregano | 0.0204 | 0.0541 |  |  |
|  | Salt | - | - |  |  |
|  |  |  |  |  |  |
| **Chaat Masala** | Cumin Seed | 13.40483 | 100.5362 | 355 | (Batra,2019q) |
|  |  |  |  |  |  |
|  | Fennel Seed | 6.702413 | 23.12332 |  |  |
|  | Garam Masala | 6.143878 | 13.6 |  |  |
|  | Mango Powder | 67.02413 | 213.807 |  |  |
|  | Black Salt | - | - |  |  |
|  | Cayenne Pepper | 4.021448 | 12.77927 |  |  |
|  | Asafoetida Powder | 1.117069 | 3.317694 |  |  |
|  | Ginger | 1.117069 | 0.893655 |  |  |
|  |  |  |  |  |  |
| **Rock Sugar** | White Sugar | 7.41 | 28.5 | 426 | (Notter et al., 2012) |
|  | Water | - | - |  |  |
|  | Royal Icing | 3.70 | 397 |  |  |
|  |  |  |  |  |  |
| **Compounded Asafoetida Powder** | Asafoetida Powder | 50.0 | 149 | 332 | (Degenhardt, 2012; Pearson, 1910) |
|  | Rice Flour | 50.0 | 183 |  |  |
|  |  |  |  |  |  |
| **Doughnut** | All Purpose Flour | 53.7 | 195 | 401 | (Reynolds, 2013) |
|  | Salt | - | - |  |  |
|  | Sourdough Starter | 44.4 | 198 |  |  |
|  | White Sugar | 2.10 | 8.09 |  |  |
|  | Water | - | - |  |  |
|  |  |  |  |  |  |
