# Supplementary material for: Biodiversity footprints of 151 popular dishes from around the world
Source: PLoS One. 2024 Feb 21;19(2):e0296492. doi: 10.1371/journal.pone.0296492 (PMC10880993; doi:10.1371/journal.pone.0296492)
Supplement: S7 Table — Chicken and eggs were assumed to have the same feed. Forage was assumed to be wheat and alfalfa for dairy cattle and lamb respectively. (DOCX) [file pone.0296492.s007.docx]

| **Livestock ingredient** | **Feed required** | **Conversion ratio** |  |
| --- | --- | --- | --- |
| **Beef** | Soymeal | 0.696 | (LCAfood-conference, 2007b) |
|  | Rapeseed oil | 0.152 |  |
|  | Wheat | 2.87 |  |
|  | Rye | 0.732 |  |
|  |  |  |  |
| **Chicken and Egg*** | Soybean | 0.827 | (LCAfood-conference, 2007c) |
|  | Rapeseed oil | 0.148 |  |
|  | Wheat | 1.44 |  |
|  |  |  |  |
| **Pork** | Wheat | 0.580 | (LCAfood-conference, 2007d) |
|  | Rapeseed oil | 0.178 |  |
|  | Soybean | 0.996 |  |
|  |  |  |  |
| **Goat** | Hay | 12.6 | (Moore et al., 2002; Solaiman, 2010) |
|  | Soybean meal | 0.717 |  |
|  |  |  |  |
| **Milk** | Grain | 0.400 | (Baldwin, 2011) |
|  | Forage (wheat) | 1.80 |  |
|  |  |  |  |
| **Lamb** | Grain | 21.0 | (Baldwin, 2011) |
|  | Forage (Alfalfa) | 30.0 |  |
