# Supplementary material for: Biodiversity footprints of 151 popular dishes from around the world
Source: PLoS One. 2024 Feb 21;19(2):e0296492. doi: 10.1371/journal.pone.0296492 (PMC10880993; doi:10.1371/journal.pone.0296492)
Supplement: S8 Table — (DOCX) [file pone.0296492.s008.docx]

| **Livestock ingredient** | **Grazing area required to raise an individual of the respective animal (in ha)** | | **Country which the area was obtained from** |
| --- | --- | --- | --- |
| **Beef** | 6.67 | (Wint, 2007) | Africa (Toit, 2014) |
| **Milk** | 2.86 | (Wint, 2007) | India (Mani, 2021) |
| **Chicken** | 0.0267 | (Robinson 2014) | China (Wint, 2007) |
| **Pork** | 0.571 | (Wint, 2007) | China (Wint, 2007) |
| **Lamb** | 1.33 | (Wint, 2007) | Near East (Wint, 2007) |
| **Egg** | 0.0267 | (TRobinson 2014) | China (Wint, 2007) |
| **Goat** | 2.86 | (Wint, 2007) | Africa (Wint, 2007) |
