# Supplementary material for: Biodiversity footprints of 151 popular dishes from around the world
Source: PLoS One. 2024 Feb 21;19(2):e0296492. doi: 10.1371/journal.pone.0296492 (PMC10880993; doi:10.1371/journal.pone.0296492)
Supplement: S9 Table — (DOCX) [file pone.0296492.s009.docx]

| **Livestock** | **Carcass weight (kg)** |  |
| --- | --- | --- |
| Cattle | 185 | (Food and Agriculture Organization of the United Nations, 2022) |
| Lamb | 14 | (Food and Agriculture Organization of the United Nations, 2022) |
| Goat | 12 | (Food and Agriculture Organization of the United Nations, 2022) |
| Pig | 50 | (Food and Agriculture Organization of the United Nations, 2022) |
| Chicken | 0.8 | (Food and Agriculture Organization of the United Nations, 2022) |
