# Supplementary material for: Biodiversity footprints of 151 popular dishes from around the world
Source: PLoS One. 2024 Feb 21;19(2):e0296492. doi: 10.1371/journal.pone.0296492 (PMC10880993; doi:10.1371/journal.pone.0296492)
Supplement: S10 Table — (DOCX) [file pone.0296492.s010.docx]

| **Food product** | **Oil absorbed during frying** | |
| --- | --- | --- |
| Potato chip | 0.400 | (Bouchon, 2009) |
| Doughnut (churros) | 0.150 | (Bouchon, 2009) |
| Papadum | 0.400 | (Bouchon, 2009) |
| Fries (triple cooked chips) | 0.150 | (Pedreschi, 2009) |
| Chickpea flour products (bonda, pakora and medu vada) | 0.452 | (Habeebrakuman et al., 2019) |
