# Supplementary material for: Biodiversity footprints of 151 popular dishes from around the world
Source: PLoS One. 2024 Feb 21;19(2):e0296492. doi: 10.1371/journal.pone.0296492 (PMC10880993; doi:10.1371/journal.pone.0296492)
Supplement: S11 Table — The dishes are ranked according to species richness indicator. (DOCX) [file pone.0296492.s011.docx]

|  |  |  |  | **Locally produced scenario** | | | **Globally produced scenario** | | |
| --- | --- | --- | --- | --- | --- | --- | --- | --- | --- |
| **Rank** | **Country of Origin** | **Diet** | **Dish** | **Species richness** | **Threatened species richness** | **Range rarity** | **Species richness** | **Threatened species richness** | **Range rarity** |
| 1 | Spain | Non-vegetarian | Lechazo | 0.286 | 0.00682 | 2.00E-09 | 0.164 | 0.00111 | 3.31E-10 |
| 2 | Brazil | Non-vegetarian | Picanha | 0.158 | 0.00626 | 8.68E-10 | 0.0342 | 0.00143 | 1.05E-10 |
| 3 | Brazil | Non-vegetarian | Fraldinha | 0.151 | 0.00599 | 8.27E-10 | 0.0330 | 0.00139 | 1.02E-10 |
| 4 | Brazil | Non-vegetarian | Churrasco | 0.140 | 0.00552 | 7.69E-10 | 0.0302 | 0.00128 | 9.37E-11 |
| 5 | Brazil | Non-vegetarian | Arroz carreteiro | 0.0947 | 0.00359 | 5.01E-10 | 0.0205 | 0.00107 | 8.23E-11 |
| 6 | India | Vegan | Idli | 0.0683 | 0.00702 | 3.37E-10 | 0.0673 | 0.00550 | 3.69E-10 |
| 7 | India | Vegan | Rajma | 0.0677 | 0.00731 | 2.98E-10 | 0.0632 | 0.00445 | 3.36E-10 |
| 8 | South Korea | Non-vegetarian | Yukgaejang | 0.0569 | 0.00332 | 3.15E-10 | 0.0480 | 0.00202 | 1.50E-10 |
| 9 | India | Vegan | Chutney | 0.0566 | 0.00601 | 2.74E-10 | 0.0485 | 0.00406 | 1.99E-10 |
| 10 | India | Non-vegetarian | Chicken chaat | 0.0469 | 0.00548 | 1.93E-10 | 0.0258 | 0.00108 | 9.45E-11 |
| 11 | Mexico | Non-vegetarian | Salsa verde pork | 0.0450 | 0.00057 | 4.60E-10 | 0.0276 | 0.00108 | 1.10E-10 |
| 12 | Mexico | Non-vegetarian | Cabrito (goat) | 0.0404 | 0.000480 | 3.39E-10 | 0.0277 | 0.00100 | 1.14E-10 |
| 13 | Mexico | Non-vegetarian | Caldo de queso | 0.0402 | 0.000381 | 9.49E-10 | 0.0475 | 0.00193 | 1.32E-10 |
| 14 | Brazil | Non-vegan | Quindim | 0.0389 | 0.00145 | 1.92E-10 | 0.0130 | 0.000758 | 1.28E-10 |
| 15 | Sweden | Vegan | Blueberry soup | 0.0362 | 0.00000 | 5.36E-11 | 0.0114 | 0.000115 | 3.04E-11 |
| 16 | United States of America | Non-vegetarian | Green chile stew | 0.0355 | 0.00143 | 1.01E-10 | 0.0384 | 0.00161 | 1.19E-10 |
| 17 | South Korea | Non-vegan | Gyeran mari | 0.0344 | 0.00185 | 1.68E-10 | 0.0352 | 0.00145 | 1.29E-10 |
| 18 | Mexico | Non-vegetarian | Caldo de pollo | 0.0339 | 0.000401 | 4.46E-10 | 0.0241 | 0.00101 | 8.91E-11 |
| 19 | India | Non-vegetarian | Chicken jalfrezi | 0.0336 | 0.00391 | 1.45E-10 | 0.0194 | 0.00100 | 7.58E-11 |
| 20 | India | Vegan | Dal | 0.0314 | 0.00313 | 1.49E-10 | 0.0242 | 0.00191 | 1.36E-10 |
| 21 | India | Vegan | Pesara | 0.0311 | 0.00338 | 1.57E-10 | 0.0358 | 0.00295 | 1.62E-10 |
| 22 | India | Vegan | Chana masala | 0.0306 | 0.00342 | 1.46E-10 | 0.0311 | 0.00304 | 1.40E-10 |
| 23 | United States of America | Non-vegetarian | Chili con carne | 0.0290 | 0.00117 | 9.13E-11 | 0.136 | 0.00913 | 6.86E-10 |
| 24 | Mexico | Non-vegetarian | Arroz verde | 0.0271 | 0.000375 | 3.71E-10 | 0.0190 | 0.000950 | 8.05E-11 |
| 25 | Spain | Non-vegan | Leche frita | 0.0266 | 0.000620 | 1.87E-10 | 0.0299 | 0.000620 | 8.75E-11 |
| 26 | Mexico | Non-vegetarian | Pork chalupas | 0.0261 | 0.000187 | 2.69E-10 | 0.0317 | 0.00193 | 1.53E-10 |
| 27 | Russia | Non-vegetarian | Pelmeni | 0.0255 | 0.000912 | 3.30E-11 | 0.0266 | 0.00110 | 8.90E-11 |
| 28 | Turkey | Non-vegan | Mahalabia | 0.0251 | 0.00128 | 9.38E-11 | 0.0246 | 0.00108 | 7.30E-11 |
| 29 | Poland | Non-vegetarian | Tatar | 0.0246 | 0.00021 | 4.09E-11 | 0.0400 | 0.00166 | 1.30E-10 |
| 30 | India | Vegan | Pakora | 0.0234 | 0.00276 | 1.09E-10 | 0.0165 | 0.00123 | 7.67E-11 |
| 31 | India | Vegan | Basmati | 0.0232 | 0.00227 | 1.15E-10 | 0.0204 | 0.00157 | 1.30E-10 |
| 32 | Spain | Non-vegan | Arroz con leche | 0.0232 | 0.00052 | 1.61E-10 | 0.0262 | 0.00107 | 8.02E-11 |
| 33 | India | Vegan | Samosa | 0.0225 | 0.00253 | 1.04E-10 | 0.0218 | 0.00224 | 9.90E-11 |
| 34 | United States of America | Non-vegetarian | Hamburger | 0.0220 | 0.000894 | 6.18E-11 | 0.0239 | 0.00100 | 7.27E-11 |
| 35 | India | Vegan | Medu vada | 0.0218 | 0.00244 | 1.03E-10 | 0.0207 | 0.00164 | 9.48E-11 |
| 36 | Poland | Non-vegetarian | Kotlety z piersi kurczaka | 0.0208 | 0.000275 | 3.47E-11 | 0.0334 | 0.00138 | 1.22E-10 |
| 37 | India | Vegan | Papadum | 0.0193 | 0.00196 | 8.28E-11 | 0.0114 | 0.000955 | 5.12E-11 |
| 38 | India | Vegan | Garam masala | 0.0183 | 0.00193 | 9.02E-11 | 0.0151 | 0.00151 | 6.85E-11 |
| 39 | Poland | Non-vegan | Budyn | 0.0173 | 0.0000542 | 2.90E-11 | 0.0301 | 0.00122 | 8.70E-11 |
| 40 | Russia | Non-vegetarian | Kasha | 0.0166 | 0.000609 | 2.31E-11 | 0.0168 | 0.000716 | 5.44E-11 |
| 41 | Mexico | Vegan | Pico de gallo (salsa) | 0.0164 | 0.0000842 | 2.83E-10 | 0.0132 | 0.000871 | 6.54E-11 |
| 42 | India | Vegan | Sambar | 0.0163 | 0.001943 | 6.80E-11 | 0.0067 | 0.000343 | 2.45E-11 |
| 43 | India | Vegan | Coconut chutney | 0.0160 | 0.00152 | 9.22E-11 | 0.0166 | 0.00129 | 1.52E-10 |
| 44 | China | Non-vegetarian | Egg drop soup | 0.0157 | 0.00109 | 5.86E-11 | 0.0242 | 0.00101 | 8.97E-11 |
| 45 | India | Vegan | Masala dosa | 0.0156 | 0.00164 | 7.58E-11 | 0.0157 | 0.00135 | 8.13E-11 |
| 46 | Brazil | Vegan | Pé-de-moleque | 0.0153 | 0.000381 | 7.91E-11 | 0.0087 | 0.000575 | 3.13E-11 |
| 47 | Spain | Non-vegetarian | Gazpacho | 0.0151 | 0.000376 | 1.05E-10 | 0.0216 | 0.000987 | 7.79E-11 |
| 48 | Venezuela | Vegan | Casabe | 0.0148 | 0.000407 | 1.06E-10 | 0.0105 | 0.000358 | 5.59E-11 |
| 49 | United States of America | Non-vegetarian | Pot roast | 0.0145 | 0.000642 | 4.01E-11 | 0.0285 | 0.00115 | 1.14E-10 |
| 50 | China | Non-vegetarian | Galinha a africana | 0.0143 | 0.000996 | 5.32E-11 | 0.0254 | 0.00122 | 1.11E-10 |
| 51 | United States of America | Vegan | Potato chips | 0.0137 | 0.000665 | 3.89E-11 | 0.0339 | 0.000665 | 1.47E-10 |
| 52 | United States of America | Non-vegetarian | Chop suey | 0.0135 | 0.000548 | 3.92E-11 | 0.0161 | 0.000686 | 5.20E-11 |
| 53 | Spain | Vegan | Espinacas con garbanzos | 0.0123 | 0.000293 | 8.55E-11 | 0.0201 | 0.00208 | 9.24E-11 |
| 54 | Poland | Non-vegetarian | Kotlet schabowy | 0.0111 | 0.000176 | 1.84E-11 | 0.0168 | 0.000668 | 6.47E-11 |
| 55 | China | Non-vegetarian | Egg foo young | 0.0108 | 0.000742 | 3.98E-11 | 0.0166 | 0.000678 | 6.24E-11 |
| 56 | Russia | Non-vegan | Pastila | 0.0106 | 0.000446 | 1.81E-11 | 0.00633 | 0.000303 | 2.23E-11 |
| 57 | Mexico | Non-vegetarian | Flour tortilla | 0.0106 | 0.000124 | 1.52E-10 | 0.00792 | 0.000334 | 2.79E-11 |
| 58 | Australia | Vegan | Avocado toast | 0.0103 | 0.000238 | 9.44E-11 | 0.00535 | 0.000193 | 6.25E-11 |
| 59 | Spain | Non-vegetarian | Pinchitos | 0.0102 | 0.000264 | 7.17E-11 | 0.0136 | 0.000643 | 4.62E-11 |
| 60 | Thailand | Vegan | Mango sticky rice | 0.0101 | 0.000930 | 7.03E-11 | 0.00685 | 0.000652 | 1.16E-10 |
| 61 | Spain | Non-vegan | Almendrados | 0.0100 | 0.000243 | 7.01E-11 | 0.00870 | 0.000269 | 5.00E-11 |
| 62 | Sweden | Vegan | Lingonberry jam | 0.0099 | 0.000000 | 1.47E-11 | 0.00237 | 0.000227 | 1.10E-11 |
| 63 | South Korea | Non-vegetarian | Samgyupsal | 0.0098 | 0.000502 | 4.90E-11 | 0.00939 | 0.000382 | 4.01E-11 |
| 64 | Indonesia | Vegan | Ketupat | 0.00934 | 0.000567 | 1.92E-10 | 0.00672 | 0.000509 | 4.26E-11 |
| 65 | China | Non-vegetarian | Rousong | 0.00925 | 0.000624 | 3.45E-11 | 0.0159 | 0.000681 | 6.27E-11 |
| 66 | Russia | Vegan | Kissel | 0.00906 | 0.000429 | 1.21E-11 | 0.00244 | 0.0000677 | 7.33E-12 |
| 67 | Canada | Non-vegetarian | Poutine | 0.00886 | 0.000179 | 2.23E-11 | 0.00886 | 0.000179 | 3.53E-11 |
| 68 | India | Vegan | Aloo gobi | 0.00844 | 0.00111 | 5.21E-11 | 0.00713 | 0.000411 | 3.59E-11 |
| 69 | India | Vegan | Bhel puri | 0.00813 | 0.000770 | 4.52E-11 | 0.00747 | 0.000596 | 6.85E-11 |
| 70 | India | Vegan | Puri | 0.00813 | 0.000770 | 4.52E-11 | 0.00747 | 0.000596 | 6.85E-11 |
| 71 | Mexico | Vegan | Mojo de ajo | 0.00803 | 0.0000715 | 1.04E-10 | 0.0095 | 0.000831 | 4.72E-11 |
| 72 | Germany | Non-vegan | Reibekuchen | 0.00764 | 0.000000 | 1.41E-11 | 0.0234 | 0.000952 | 8.23E-11 |
| 73 | Japan | Non-vegetarian | Chicken sashimi | 0.00764 | 0.000132 | 1.04E-10 | 0.00935 | 0.000370 | 3.63E-11 |
| 74 | Poland | Non-vegan | Kogel mogel | 0.00756 | 0.000100 | 1.26E-11 | 0.0121 | 0.000501 | 4.42E-11 |
| 75 | Spain | Non-vegan | Migas | 0.00756 | 0.000215 | 5.71E-11 | 0.0125 | 0.000523 | 4.59E-11 |
| 76 | Spain | Non-vegan | Tarta de santiago | 0.00743 | 0.000191 | 5.28E-11 | 0.00799 | 0.000282 | 3.88E-11 |
| 77 | India | Vegan | Putu mayam | 0.00737 | 0.000726 | 3.65E-11 | 0.00637 | 0.000485 | 4.04E-11 |
| 78 | Japan | Vegan | Edamame | 0.00688 | 0.000123 | 1.05E-10 | 0.00791 | 0.000284 | 3.47E-11 |
| 79 | Japan | Vegan | Yuba | 0.00688 | 0.000123 | 1.05E-10 | 0.00791 | 0.000284 | 3.47E-11 |
| 80 | China | Non-vegetarian | Pepper steak | 0.00680 | 0.000469 | 2.51E-11 | 0.0100 | 0.000399 | 3.64E-11 |
| 81 | Mexico | Vegan | Guacamole | 0.00679 | 0.000139 | 1.10E-10 | 0.00773 | 0.000274 | 1.21E-10 |
| 82 | China | Non-vegetarian | Tomato and egg soup | 0.00667 | 0.000457 | 2.49E-11 | 0.0102 | 0.000418 | 3.96E-11 |
| 83 | Netherlands | Non-vegan | Griesmeel pudding | 0.00644 | 0.000000 | 9.87E-12 | 0.0391 | 0.00159 | 1.05E-10 |
| 84 | South Korea | Vegan | Kongguksu | 0.00632 | 0.000318 | 3.17E-11 | 0.0096 | 0.000435 | 4.71E-11 |
| 85 | Turkey | Non-vegan | Yufka | 0.00631 | 0.000379 | 3.08E-11 | 0.00812 | 0.000354 | 2.68E-11 |
| 86 | China | Non-vegetarian | Moo shu pork | 0.00610 | 0.000411 | 2.27E-11 | 0.00955 | 0.000411 | 3.80E-11 |
| 87 | Spain | Non-vegan | Torrijas | 0.00591 | 0.000150 | 4.19E-11 | 0.00811 | 0.000355 | 2.58E-11 |
| 88 | India | Vegan | Chapati | 0.00588 | 0.000668 | 2.28E-11 | 0.00387 | 0.000180 | 1.09E-11 |
| 89 | Italy | Vegan | Farinata di ceci | 0.00587 | 0.000155 | 2.77E-11 | 0.0203 | 0.00210 | 9.40E-11 |
| 90 | India | Vegan | Naan | 0.00580 | 0.000672 | 3.30E-11 | 0.00380 | 0.000169 | 1.02E-11 |
| 91 | China | Non-vegetarian | Kung pao chicken | 0.00557 | 0.000379 | 2.05E-11 | 0.00880 | 0.000361 | 3.44E-11 |
| 92 | Spain | Non-vegan | Arroz con huevo | 0.00542 | 0.000161 | 3.97E-11 | 0.0155 | 0.000959 | 8.14E-11 |
| 93 | China | Vegan | Black sesame soup | 0.00541 | 0.000376 | 2.14E-11 | 0.0356 | 0.00232 | 1.45E-10 |
| 94 | China | Non-vegetarian | Lo mein | 0.00532 | 0.000362 | 1.96E-11 | 0.00810 | 0.000322 | 3.15E-11 |
| 95 | Saudi Arabia | Vegan | Hummus | 0.00526 | 0.000358 | 3.57E-11 | 0.0314 | 0.00278 | 1.33E-10 |
| 96 | India | Vegan | Aloo paratha | 0.00495 | 0.000561 | 2.09E-11 | 0.00358 | 0.000174 | 1.13E-11 |
| 97 | Poland | Non-vegan | Silesian kluski | 0.00491 | 0.000037 | 8.27E-12 | 0.00804 | 0.000356 | 2.47E-11 |
| 98 | Turkey | Vegan | Pide | 0.00480 | 0.000249 | 1.80E-11 | 0.00571 | 0.000275 | 1.66E-11 |
| 99 | United States of America | Vegan | Peanut butter sandwich | 0.00472 | 0.000153 | 2.53E-11 | 0.0105 | 0.000643 | 3.62E-11 |
| 100 | France | Vegan | Ratatouille | 0.00459 | 0.00000217 | 1.85E-11 | 0.0123 | 0.000846 | 6.45E-11 |
| 101 | United States of America | Non-vegetarian | Chicken sandwich | 0.00453 | 0.000184 | 1.27E-11 | 0.00593 | 0.000272 | 1.90E-11 |
| 102 | Poland | Non-vegan | Kopytkami | 0.00446 | 0.0000330 | 7.49E-12 | 0.00751 | 0.000332 | 2.34E-11 |
| 103 | India | Vegan | Dosa | 0.00444 | 0.000478 | 1.96E-11 | 0.00324 | 0.000204 | 2.33E-11 |
| 104 | Germany | Non-vegan | Kartoffelpuffer | 0.00443 | 0.000000 | 8.17E-12 | 0.0135 | 0.000562 | 5.13E-11 |
| 105 | China | Non-vegan | Scallion pancake | 0.00436 | 0.000296 | 1.60E-11 | 0.00662 | 0.000260 | 2.59E-11 |
| 106 | Turkey | Vegan | Ramazan pidesi | 0.00423 | 0.000220 | 1.59E-11 | 0.00432 | 0.000193 | 1.17E-11 |
| 107 | United States of America | Vegan | Sourdough bread | 0.00410 | 0.000164 | 1.14E-11 | 0.00407 | 0.000181 | 1.09E-11 |
| 108 | Italy | Vegan | Penne all’arrabbiata | 0.00387 | 0.0000755 | 2.18E-11 | 0.00886 | 0.000390 | 3.08E-11 |
| 109 | India | Vegan | Bonda | 0.00380 | 0.000411 | 1.80E-11 | 0.00394 | 0.000332 | 1.64E-11 |
| 110 | Poland | Non-vegan | Pyzy | 0.00378 | 0.0000266 | 6.37E-12 | 0.00620 | 0.000276 | 1.87E-11 |
| 111 | South Korea | Vegan | Gamjajeon | 0.00371 | 0.000177 | 1.82E-11 | 0.00542 | 0.000260 | 1.43E-11 |
| 112 | Belgium | Non-vegan | Stoemp | 0.00355 | 0.00000 | 6.61E-12 | 0.01091 | 0.000487 | 2.92E-11 |
| 113 | United States of America | Vegan | Sicilian pizza | 0.00335 | 0.000131 | 1.17E-11 | 0.00387 | 0.000182 | 1.23E-11 |
| 114 | Spain | Vegan | Pan de barra | 0.00334 | 0.0000736 | 2.27E-11 | 0.00389 | 0.000173 | 1.04E-11 |
| 115 | China | Vegan | Shao bing | 0.00283 | 0.000204 | 1.01E-11 | 0.0112 | 0.000650 | 3.91E-11 |
| 116 | Japan | Vegan | Udon | 0.00279 | 0.0000450 | 2.64E-11 | 0.00393 | 0.000183 | 1.11E-11 |
| 117 | Spain | Non-vegan | Panellets | 0.00273 | 0.0000664 | 1.90E-11 | 0.00280 | 0.000097 | 1.40E-11 |
| 118 | United States of America | Non-vegan | Fortune cookie | 0.00269 | 0.000113 | 7.43E-12 | 0.00421 | 0.000175 | 1.54E-11 |
| 119 | France | Vegan | Pistou | 0.00256 | 0.0000280 | 4.85E-11 | 0.00734 | 0.000372 | 5.63E-11 |
| 120 | Spain | Vegan | Escalivada | 0.00251 | 0.0000647 | 1.78E-11 | 0.00765 | 0.0000647 | 3.90E-11 |
| 121 | Spain | Vegan | Quince paste | 0.00241 | 0.0000573 | 1.67E-11 | 0.00249 | 0.000142 | 1.14E-11 |
| 122 | China | Vegan | Man tou | 0.00235 | 0.00017 | 8.43E-12 | 0.00381 | 0.000169 | 1.02E-11 |
| 123 | Germany | Vegan | Rote grütze | 0.00228 | 0.00000 | 4.09E-12 | 0.00313 | 0.0000458 | 8.55E-12 |
| 124 | Italy | Vegan | Pizza romana | 0.00213 | 0.0000378 | 9.51E-12 | 0.00437 | 0.000215 | 1.39E-11 |
| 125 | Italy | Vegan | Grissini | 0.00213 | 0.0000425 | 9.74E-12 | 0.02202 | 0.00134 | 7.91E-11 |
| 126 | Spain | Vegan | Sofrito | 0.00208 | 0.0000552 | 1.49E-11 | 0.00548 | 0.000320 | 2.71E-11 |
| 127 | Spain | Vegan | Pisto | 0.00205 | 0.0000556 | 1.48E-11 | 0.00666 | 0.000411 | 3.65E-11 |
| 128 | Spain | Vegan | Porrusalda | 0.00190 | 0.0000470 | 1.32E-11 | 0.00383 | 0.000241 | 2.11E-11 |
| 129 | Japan | Vegan | Yaki-imo | 0.00187 | 0.0000288 | 2.72E-11 | 0.00341 | 0.000164 | 8.04E-12 |
| 130 | Belgium | Vegan | Sirop de liège | 0.00186 | 0.00000 | 3.50E-12 | 0.00751 | 0.000413 | 2.71E-11 |
| 131 | China | Vegan | Mala sauce | 0.00182 | 0.000134 | 7.13E-12 | 0.0170 | 0.00126 | 7.14E-11 |
| 132 | Spain | Vegan | Papas arrugadas | 0.00181 | 0.0000327 | 1.03E-11 | 0.00435 | 0.000209 | 1.20E-11 |
| 133 | Germany | Vegan | Apfelmus | 0.00177 | 0.00000 | 3.18E-12 | 0.00764 | 0.000408 | 2.62E-11 |
| 134 | Spain | Vegan | Pa amb tomàquet | 0.00170 | 0.0000373 | 1.16E-11 | 0.00209 | 0.000096 | 5.86E-12 |
| 135 | China | Vegan | Nuomici | 0.00160 | 0.000118 | 6.87E-12 | 0.00580 | 0.000473 | 7.28E-11 |
| 136 | South Korea | Vegan | Dotorimuk | 0.00152 | 0.0000727 | 7.47E-12 | 0.00611 | 0.000281 | 4.84E-11 |
| 137 | Italy | Vegan | Ciabatter | 0.00147 | 0.0000257 | 6.81E-12 | 0.00269 | 0.000122 | 7.40E-12 |
| 138 | Switzerland | Vegan | Walliser roggenbrot | 0.00136 | 0.00000 | 4.33E-12 | 0.00428 | 0.000149 | 8.67E-12 |
| 139 | Switzerland | Vegan | St. Galler brot | 0.00125 | 0.00000 | 3.82E-12 | 0.00386 | 0.000171 | 1.03E-11 |
| 140 | France | Vegan | Baguette | 0.00119 | 0.00000 | 3.02E-12 | 0.00473 | 0.000220 | 1.33E-11 |
| 141 | Germany | Vegan | Pellkartoffeln | 0.00113 | 0.00000 | 2.01E-12 | 0.00529 | 0.000254 | 1.25E-11 |
| 142 | France | Non-vegan | Macarons | 0.00113 | 0.00000 | 2.78E-12 | 0.00454 | 0.000171 | 1.99E-11 |
| 143 | Japan | Vegan | Warabi mochi | 0.000987 | 0.0000173 | 1.50E-11 | 0.00120 | 0.0000454 | 4.88E-12 |
| 144 | Spain | Vegan | Tomato frito | 0.000986 | 0.0000326 | 7.53E-12 | 0.00239 | 0.000135 | 1.18E-11 |
| 145 | Sweden | Vegan | Mjukkaka | 0.000876 | 0.00000 | 1.34E-12 | 0.00323 | 0.000148 | 9.05E-12 |
| 146 | United Kingdom | Vegan | Triple cooked chips | 0.000717 | 0.00000 | 1.44E-12 | 0.00191 | 0.0000866 | 5.25E-12 |
| 147 | Spain | Vegan | Pimientos de padron | 0.000688 | 0.0000222 | 5.17E-12 | 0.00266 | 0.000140 | 1.41E-11 |
| 148 | United States of America | Vegan | Popcorn | 0.000608 | 0.0000226 | 2.64E-12 | 0.00233 | 0.0000748 | 1.57E-11 |
| 149 | Italy | Vegan | Polenta | 0.000490 | 0.00000 | 1.37E-12 | 0.00540 | 0.000171 | 3.81E-11 |
| 150 | United Kingdom | Vegan | Crumpet | 0.000481 | 0.00000 | 9.71E-13 | 0.00388 | 0.000170 | 1.03E-11 |
| 151 | Belgium | Vegan | Pommes frites | 0.000431 | 0.00000 | 1.04E-12 | 0.00169 | 0.0000609 | 7.30E-12 |
