# Supplementary material for: Biodiversity footprints of 151 popular dishes from around the world
Source: PLoS One. 2024 Feb 21;19(2):e0296492. doi: 10.1371/journal.pone.0296492 (PMC10880993; doi:10.1371/journal.pone.0296492)
Supplement: S12 Table — The dishes are ranked according to species richness indicator. (DOCX) [file pone.0296492.s012.docx]

|  |  |  |  | **Locally produced scenario** | | | **Globally produced scenario** | | |
| --- | --- | --- | --- | --- | --- | --- | --- | --- | --- |
| **Rank** | **Country of origin** | **Diet** | **Dish** | **Species richness** | **Threatened species richness** | **Range rarity** | **Species richness** | **Threatened species richness** | **Range rarity** |
| 1 | Mexico | Meat dish | Cabrito (goat) | 16.0 | 1.65E-01 | 1.17E-07 | 12.22 | 0.661 | 6.09E-08 |
| 2 | Brazil | Meat dish | Picanha | 2.66 | 1.18E-01 | 1.19E-08 | 0.876 | 0.0474 | 4.37E-09 |
| 3 | Brazil | Meat dish | Fraldinha | 2.50 | 1.11E-01 | 1.11E-08 | 0.821 | 0.0444 | 4.09E-09 |
| 4 | Brazil | Meat dish | Churrasco | 2.31 | 1.03E-01 | 1.03E-08 | 0.759 | 0.0410 | 3.78E-09 |
| 5 | Spain | Meat dish | Lechazo | 1.82 | 1.22E-01 | 1.06E-08 | 3.000 | 0.162 | 1.49E-08 |
| 6 | Mexico | Meat dish | Caldo de queso | 1.61 | 1.66E-02 | 1.18E-08 | 1.23 | 0.0664 | 6.12E-09 |
| 7 | India | Meat dish | Chicken chaat | 1.54 | 1.45E-01 | 8.10E-09 | 0.895 | 0.0484 | 4.46E-09 |
| 8 | South Korea | Vegetarian | Gyeran mari | 1.31 | 6.74E-02 | 5.84E-09 | 1.25 | 0.0674 | 6.21E-09 |
| 9 | South Korea | Meat dish | Yukgaejang | 1.27 | 6.50E-02 | 5.64E-09 | 1.20 | 0.0650 | 5.99E-09 |
| 10 | Brazil | Meat dish | Arroz carreteiro | 1.12 | 4.94E-02 | 5.01E-09 | 0.365 | 0.0198 | 1.82E-09 |
| 11 | Mexico | Meat dish | Caldo de pollo | 1.10 | 1.13E-02 | 8.00E-09 | 0.837 | 0.0452 | 4.17E-09 |
| 12 | Brazil | Vegetarian | Quindim | 1.02 | 4.54E-02 | 4.57E-09 | 0.338 | 0.0185 | 1.76E-09 |
| 13 | India | Meat dish | Chicken jalfrezi | 0.927 | 8.77E-02 | 4.89E-09 | 0.541 | 0.0294 | 2.69E-09 |
| 14 | United States of America | Meat dish | Green chile stew | 0.757 | 2.61E-02 | 3.79E-09 | 0.966 | 0.0522 | 4.81E-09 |
| 15 | Poland | Meat dish | Kotlety z piersi kurczaka | 0.706 | 1.60E-02 | 1.01E-09 | 1.19 | 0.0642 | 5.91E-09 |
| 16 | Poland | Meat dish | Tatar | 0.692 | 1.57E-02 | 9.91E-10 | 1.16 | 0.0629 | 5.80E-09 |
| 17 | Mexico | Meat dish | Arroz verde | 0.679 | 7.07E-03 | 5.01E-09 | 0.518 | 0.0281 | 2.58E-09 |
| 18 | Mexico | Meat dish | Salsa verde pork | 0.522 | 5.39E-03 | 3.81E-09 | 0.399 | 0.0215 | 1.99E-09 |
| 19 | Poland | Vegetarian | Budyn | 0.493 | 1.12E-02 | 7.07E-10 | 0.830 | 0.0448 | 4.13E-09 |
| 20 | China | Meat dish | Egg drop soup | 0.490 | 4.55E-02 | 1.61E-09 | 0.842 | 0.0455 | 4.20E-09 |
| 21 | Spain | Vegetarian | Leche frita | 0.475 | 3.16E-02 | 2.75E-09 | 0.781 | 0.0422 | 3.89E-09 |
| 22 | United States of America | Meat dish | Hamburger | 0.468 | 1.61E-02 | 2.34E-09 | 0.597 | 0.0322 | 2.97E-09 |
| 23 | China | Meat dish | Galinha a africana | 0.427 | 3.97E-02 | 1.41E-09 | 0.737 | 0.0400 | 3.69E-09 |
| 24 | Netherlands | Vegetarian | Griesmeel pudding | 0.397 | 1.28E-02 | 4.83E-10 | 0.950 | 0.0513 | 4.73E-09 |
| 25 | Turkey | Vegetarian | Mahalabia | 0.388 | 3.88E-02 | 1.43E-09 | 0.575 | 0.0312 | 2.87E-09 |
| 26 | Spain | Vegetarian | Arroz con leche | 0.363 | 2.42E-02 | 2.10E-09 | 0.599 | 0.0325 | 2.99E-09 |
| 27 | United States of America | Meat dish | Chili con carne | 0.343 | 1.19E-02 | 1.70E-09 | 0.539 | 0.0311 | 2.73E-09 |
| 28 | United States of America | Meat dish | Pot roast | 0.319 | 1.10E-02 | 1.60E-09 | 0.407 | 0.0220 | 2.03E-09 |
| 29 | Russia | Meat dish | Pelmeni | 0.313 | 2.23E-02 | 4.25E-10 | 0.550 | 0.0297 | 2.73E-09 |
| 30 | United States of America | Meat dish | Chop suey | 0.308 | 1.06E-02 | 1.54E-09 | 0.393 | 0.0213 | 1.96E-09 |
| 31 | Spain | Meat dish | Gazpacho | 0.264 | 1.75E-02 | 1.53E-09 | 0.435 | 0.0235 | 2.17E-09 |
| 32 | Poland | Vegetarian | Kogel mogel | 0.257 | 5.84E-03 | 3.68E-10 | 0.432 | 0.0233 | 2.15E-09 |
| 33 | China | Meat dish | Egg foo young | 0.255 | 2.37E-02 | 8.41E-10 | 0.438 | 0.0236 | 2.18E-09 |
| 34 | Russia | Meat dish | Kasha | 0.229 | 1.61E-02 | 3.11E-10 | 0.397 | 0.0214 | 1.96E-09 |
| 35 | Germany | Vegetarian | Reibekuchen | 0.228 | 5.90E-03 | 3.16E-10 | 0.447 | 0.0240 | 2.21E-09 |
| 36 | Germany | Vegetarian | Kartoffelpuffer | 0.220 | 5.77E-03 | 3.04E-10 | 0.428 | 0.0231 | 2.13E-09 |
| 37 | Spain | Vegetarian | Migas | 0.216 | 1.43E-02 | 1.26E-09 | 0.356 | 0.0192 | 1.77E-09 |
| 38 | Spain | Meat dish | Pinchitos | 0.180 | 1.20E-02 | 1.05E-09 | 0.296 | 0.0161 | 1.48E-09 |
| 39 | Mexico | Meat dish | Pork chalupas | 0.167 | 1.61E-03 | 1.26E-09 | 0.141 | 0.00796 | 7.06E-10 |
| 40 | Poland | Meat dish | Kotlet schabowy | 0.161 | 3.64E-03 | 2.30E-10 | 0.270 | 0.0146 | 1.34E-09 |
| 41 | Spain | Vegetarian | Arroz con huevo | 0.132 | 8.76E-03 | 7.69E-10 | 0.224 | 0.0123 | 1.13E-09 |
| 42 | South Korea | Meat dish | Samgyupsal | 0.131 | 6.70E-03 | 5.82E-10 | 0.124 | 0.00668 | 6.17E-10 |
| 43 | Japan | Meat dish | Chicken sashimi | 0.121 | 1.82E-03 | 1.15E-09 | 0.134 | 0.00718 | 6.65E-10 |
| 44 | Turkey | Vegetarian | Yufka | 0.115 | 1.14E-02 | 4.23E-10 | 0.169 | 0.00912 | 8.35E-10 |
| 45 | China | Meat dish | Tomato and egg soup | 0.0998 | 9.19E-03 | 3.30E-10 | 0.171 | 0.00916 | 8.46E-10 |
| 46 | Poland | Vegetarian | Silesian kluski | 0.0972 | 2.16E-03 | 1.40E-10 | 0.164 | 0.00882 | 8.05E-10 |
| 47 | Spain | Vegetarian | Torrijas | 0.0969 | 6.33E-03 | 5.64E-10 | 0.158 | 0.00852 | 7.80E-10 |
| 48 | Mexico | Meat dish | Flour tortilla | 0.0924 | 9.49E-04 | 7.26E-10 | 0.071 | 0.00384 | 3.49E-10 |
| 49 | Poland | Vegetarian | Kopytkami | 0.0866 | 1.92E-03 | 1.25E-10 | 0.146 | 0.00786 | 7.18E-10 |
| 50 | Spain | Vegetarian | Tarta de santiago | 0.0829 | 5.30E-03 | 4.86E-10 | 0.132 | 0.00703 | 6.61E-10 |
| 51 | United States of America | Vegetarian | Fortune cookie | 0.0809 | 2.79E-03 | 4.03E-10 | 0.103 | 0.00557 | 5.13E-10 |
| 52 | China | Meat dish | Rousong | 0.0743 | 6.82E-03 | 2.46E-10 | 0.129 | 0.00694 | 6.36E-10 |
| 53 | Belgium | Vegetarian | Stoemp | 0.0727 | 0.002096 | 1.08E-10 | 0.160 | 0.00863 | 7.86E-10 |
| 54 | Canada | Meat dish | Poutine | 0.0707 | 1.72E-03 | 2.33E-10 | 0.128 | 0.00689 | 6.35E-10 |
| 55 | Poland | Vegetarian | Pyzy | 0.0700 | 1.55E-03 | 1.01E-10 | 0.118 | 0.00635 | 5.79E-10 |
| 56 | Russia | Vegetarian | Pastila | 0.0666 | 4.54E-03 | 9.42E-11 | 0.107 | 0.00577 | 5.26E-10 |
| 57 | China | Meat dish | Pepper steak | 0.0656 | 6.00E-03 | 2.18E-10 | 0.112 | 0.00596 | 5.52E-10 |
| 58 | United States of America | Meat dish | Chicken sandwich | 0.0631 | 2.19E-03 | 3.09E-10 | 0.0802 | 0.00431 | 3.92E-10 |
| 59 | France | Vegetarian | Macarons | 0.0588 | 1.50E-03 | 1.39E-10 | 0.113 | 0.00605 | 5.63E-10 |
| 60 | China | Meat dish | Kung pao chicken | 0.0575 | 5.29E-03 | 1.90E-10 | 0.0988 | 0.00530 | 4.89E-10 |
| 61 | China | Meat dish | Lo mein | 0.0512 | 4.66E-03 | 1.70E-10 | 0.0873 | 0.00463 | 4.29E-10 |
| 62 | Spain | Vegetarian | Almendrados | 0.0322 | 1.74E-03 | 1.97E-10 | 0.0451 | 0.00225 | 2.33E-10 |
| 63 | China | Meat dish | Moo shu pork | 0.0315 | 2.83E-03 | 1.05E-10 | 0.0540 | 0.00283 | 2.62E-10 |
| 64 | China | Vegetarian | Scallion pancake | 0.0263 | 2.35E-03 | 8.80E-11 | 0.0445 | 0.00232 | 2.16E-10 |
| 65 | Spain | Vegetarian | Panellets | 0.0141 | 8.82E-04 | 8.27E-11 | 0.0223 | 0.00118 | 1.11E-10 |
