# Supplementary material for: Biodiversity footprints of 151 popular dishes from around the world
Source: PLoS One. 2024 Feb 21;19(2):e0296492. doi: 10.1371/journal.pone.0296492 (PMC10880993; doi:10.1371/journal.pone.0296492)
Supplement: S1 Text — (DOCX) [file pone.0296492.s014.docx]

**Supporting references**

Addison, K. (2021). Oil yields and characteristics. http://journeytoforever.org/biodiesel_yield.html.

Ahmad, M.M., Rehman, S.-u., Tahir, M., Qureshi, T., Nadeem, M., and Asghar, M. (2016). Variability In Peel Composition And Quality Evaluation Of Peel Oils Of Citrus Varieties

Alderson, E. (2014). The Homemade Flour Cookbook (Fair Winds Press).

Amy (2021). Sourdough country loaf. https://littlespoonfarm.com/sourdough-country-loaf-recipe/.

Arena, A., Bisignano, C., Stassi, G., Mandalari, G., Wickham, M.S.J., and Bisignano, G. (2010). Immunomodulatory and antiviral activity of almond skins. Immunology Letters 132 (1), 18-23.

Aziz, N.S., Sofian-Seng, N.-S., Mohd Razali, N.S., Lim, S.J., and Mustapha, W.A. (2019). A review on conventional and biotechnological approaches in white pepper production. Journal of the Science of Food and Agriculture 99 (6), 2665-2676.

Baldwin, C.J. (2011). Sustainability in the Food Industry (Wiley).

Bensebia, O., and Allia, K. (2016). Analysis of adsorption–desorption moisture isotherms of rosemary leaves. Journal of Applied Research on Medicinal and Aromatic Plants 3 (3), 79-86.

Beranbaum, R.L. (2003). The Bread Bible (W. W. Norton & Company).

Bergthaller, W., Witt, W., and Goldau, H.-P. (1999). Potato Starch Technology. Starch - Stärke 51 (7), 235-242.

Beyer, R., and A. Manica. 2021. Global and country-level data of the biodiversity footprints of 175 crops and pasture. Data in Brief 36:106982.

Bill, J., Sarah, Kaitlin (2019). Dark Soy Sauce. https://thewoksoflife.com/dark-soy-sauce/.

Chetachukwu, A.S., Thongraung, C., and Yupanqui, C.T. (2018). Effect of short-chain inulin on the rheological and sensory characteristics of reduced fat set coconut milk yoghurt. Journal of Texture Studies 49 (4), 434-447.

Cook.me Inc (2019). Salt Pork. https://cook.me/recipe/salt-pork/.

Dalgaard, R., Schmidt, J., Halberg, N., Christensen, P., Thrane, M., and Pengue, W.A. (2007). LCA of soybean meal. The International Journal of Life Cycle Assessment 13 (3), 240.

Degenhardt, M.L., Birgit Kohlenberg, Beate Hartmann, Michael Roloff, Stefan Brennecke, Laurence Guibouret, Berthold Weber, Gerhard Krammer (2012). Novel Insights into Flavor Chemistry of Asafetida. American Chemical Society.

Delish (2022) Raspberry Jam. https://www.delish.com/cooking/recipe-ideas/a27757299/easy-raspberry-jam-recipe/

Dongre, R.A. (2020). Vegan Sourdough Burger Buns. https://cookwithrenu.com/vegan-sourdough-burger-buns-breadbakers/.

Figiel, A., Szumny, A., Gutiérrez-Ortíz, A., and Carbonell-Barrachina, Á.A. (2010). Composition of oregano essential oil (Origanum vulgare) as affected by drying method. Journal of Food Engineering 98 (2), 240-247.

Gaston O. Adoyo, D.N.S., Arnold N. Onyango (2021). Physico-chemical properties of kernel from coconut (Cocos nucifera L.) varieties grown at the Kenyan Coast. African Journal of Food Science 15 (8), 313-321.

Hadjiandreou, E. (2016). How To Make Sourdough: 45 recipes for great-tasting sourdough breads that are good for you, too (Ryland Peters & Small).

Hirun, S., Utama-ang, N., and Roach, P.D. (2014). Turmeric (Curcuma longa L.) drying: an optimization approach using microwave-vacuum drying. Journal of Food Science and Technology 51 (9), 2127-2133.

Inglett, G. (2012). Tropical Food: Chemistry and Nutrition V2 (Elsevier Science).

Joachim, D., and Hoffman, M. (2000). Prevention's The Healthy Cook: The Ultimate Illustrated Kitchen Guide to Great Low-Fat Food (Rodale Books).

Judy (2020). Homemade Rice Noodle. https://thewoksoflife.com/homemade-rice-noodles/.

Kang, M., and Priyadarshan, P.M. (2008). Breeding Major Food Staples (Wiley).

Kumar, C.S., and Bhattacharya, S. (2008). Tamarind Seed: Properties, Processing and Utilization. Critical Reviews in Food Science and Nutrition 48 (1), 1-20.

Kumar, R., Sahay, S., Mishra, P., and Rashmi, K. (2015). Effect of Nitrogen Phosphorus and Potash on Coriander Yield.

LCAfood-conference (2007a). Flour and oat flakes. http://www.lcafood.dk/.

LCAfood-conference (2007b). Living cattle ex farm. http://www.lcafood.dk/.

LCAfood-conference (2007c). Living chicken ex farm. http://www.lcafood.dk/.

LCAfood-conference (2007d). Living pig ex farm. http://www.lcafood.dk/.

LCAfood-conference (2007e). Sugar (sukker). http://www.lcafood.dk/.

LCAfood-conference (2007f). Vegetable oil. http://www.lcafood.dk/.

Marshall, C. (2021). Bread Flour Substitute: Can You Use All Purpose Flour?

Martin, M.E., Grao-Cruces, E., Millan-Linares, M.C., and Montserrat-de la Paz, S. (2020). Grape (Vitis vinifera L.) Seed Oil: A Functional Food from the Winemaking Industry. Foods 9 (10), 1360.

Mike Foale, H.H. (2011). Farm and Forestry Production and Marketing Profile for Coconut. http://agroforestry.net/scps.

Moon, H.R., Chung, M.J., Park, J.W., Cho, S.M., Choi, D.J., Kim, S.M., Chun, M.H., Kim, I.-B., Kim, S.O., Jang, S.J., and Park, Y.I. (2013). Antiasthma effects through anti-inflammatory action of acorn (quercus acutissima carr.) In vitro and in vivo. Journal of Food Biochemistry 37 (1), 108-118.

Morton, J. (2019). Super Sourdough: The Foolproof Guide to Making World-Class Bread at Home (Quadrille).

Moufida, S.d., and Marzouk, B. (2003). Biochemical characterization of blood orange, sweet orange, lemon, bergamot and bitter orange. Phytochemistry 62 (8), 1283-1289.

Notter, E., Brooks, J., and Schaeffer, L. (2012). The Art of the Confectioner: Sugarwork and Pastillage (Wiley).

Nyonya Cooking (2021). Homemade Wheat Noodles (without Egg). https://www.nyonyacooking.com/recipes/homemade-wheat-noodles-without-egg~ByCEOwivz5-Q.

Ojakangas, B. (2020). The Soup and Bread Cookbook (University of Minnesota Press).

Özcan, M., Arslan, D., and Ünver, A. (2005). Effect of drying methods on the mineral content of basil (Ocimum basilicum L.). Journal of Food Engineering 69 (3), 375-379.

Pearson, W.A. (1910). Asafetida. Journal of Industrial & Engineering Chemistry 2 (10), 421-423.

Pinoy, P. (2021). How to Make Soy Sauce. https://panlasangpinoy.com/how-to-make-soy-sauce/.

Rai, R. (2000). Curry, Curry, Curry (Penguin Books Limited).

Raichlen, S. (2010). Planet Barbecue!: 309 Recipes, 60 Countries (Workman Publishing Company).

Rema, J., and Krishnamoorthy, B. (2012). 22 - Nutmeg and mace. In Handbook of Herbs and Spices (Second Edition), K.V. Peter, ed. (Woodhead Publishing), pp. 399-416.

Reynolds, R. (2013). Doughnuts: A Classic Treat Reinvented – 60 easy, delicious recipes (Ebury Publishing).

Sakhale, B., Nandane, A., and Ranveer, D.R. (2007). Studies On Dehydration Of Curry Leaves. Adit J of Engg. 4, 62-64.

Samira (2020). How To Make Garlic Powder. https://www.alphafoodie.com/how-to-make-garlic-powder/.

Samira (2021). How to Make Cornmeal. https://www.alphafoodie.com/how-to-make-cornmeal/.

Sayaslan, A. (2004). Wet-milling of wheat flour: industrial processes and small-scale test methods. LWT - Food Science and Technology 37 (5), 499-515.

Scherer, L., and Pfister, S. (2016). Global Biodiversity Loss by Freshwater Consumption and Eutrophication from Swiss Food Consumption. Environmental Science & Technology 50 (13), 7019-7028.

Siles, J.A., González-Tello, P., Martín, M.A., and Martín, A. (2015). Kinetics of alfalfa drying: Simultaneous modelling of moisture content and temperature. Biosystems Engineering 129, 185-196.

Solomon, K., and Martin, J. (2009). Jam It, Pickle It, Cure It: And Other Cooking Projects (Ten Speed Press).

Soysal, Y. (2004). Microwave Drying Characteristics of Parsley. Biosystems Engineering 89 (2), 167-173.

Srivastava, K.C. (1993). Antiplatelet principles from a food spice clove (Syzgium aromaticum L). Prostaglandins, Leukotrienes and Essential Fatty Acids 48 (5), 363-372.

Stafford, G. (2020). How to Make Breadcrumbs. https://www.biggerbolderbaking.com/how-to-make-breadcrumbs/.

Suleiman, R., Rosentrater, K.A., and Bern, C.J. (2015). Evaluation of maize weevils Sitophilus zeamais Motschulsky infestation on seven varieties of maize. Journal of Stored Products Research 64, 97-102.

Thirst Quenching Lime. (1983). Nutrition & Food Science 83 (2), 15-16.

Thomas, H. (2018). The Nut Butter Cookbook, 1st Edition (HarperCollins Publishers).

Tsao, C.-H., Chang, C.-W., Ho, Y.-C., Chuang, Y.-K., and Lee, W.-J. (2021). Application of OXITEST for Prediction of Shelf-Lives of Selected Cold-Pressed Oils. Frontiers in Nutrition 8 (808).

U.S. Department of Agriculture (1992). Weights, Measures, and Conversion Factors for Agricultural Commodities and Their Products. https://www.ers.usda.gov/webdocs/publications/41880/33132_ah697_002.pdf.

U.S. Department of Agriculture (2021a). FoodData Central Search Results. https://fdc.nal.usda.gov/fdc-app.html#/

Varma, A.K., Singh, S., Rathore, A.K., Thakur, L.S., Shankar, R., and Mondal, P. (2020). Investigation of kinetic and thermodynamic parameters for pyrolysis of peanut shell using thermogravimetric analysis. Biomass Conversion and Biorefinery.

Velasco-Arango, V.A., Bernal-Martínez, A.A., Ordóñez-Santos, L.E., and Hleap-Zapata, J.I. (2020). Characterization of the epicarp of guava ( Psidium guajava L.) as a natural alternative for use in processed food products. Biotechnology in the Agricultural and Agroindustrial Sector 18, 26-35.

Vossen, P. (2007). Olive Oil: History, Production, and Characteristics of the World's Classic Oils. HortScience horts 42 (5), 1093-1100.

Walker, H. (1993). Look and Feel: Studies in Texture, Appearance and Incidental Characteristics of Food : Proceedings of the Oxford Symposium on Food and Cookery (Oxford Symposium).

Yuttitham, M., Gheewala, S.H., and Chidthaisong, A. (2011). Carbon footprint of sugar produced from sugarcane in eastern Thailand. Journal of Cleaner Production 19 (17), 2119-2127.
